# Degradation of GSPT1 causes TP53-independent cell death in leukemia while sparing normal hematopoietic stem cells

**DOI:** 10.1172/JCI153514

**Published:** 2022-08-15

**Authors:** Rob S. Sellar, Adam S. Sperling, Mikołaj Słabicki, Jessica A. Gasser, Marie E. McConkey, Katherine A. Donovan, Nada Mageed, Dylan N. Adams, Charles Zou, Peter G. Miller, Ravi K. Dutta, Steffen Boettcher, Amy E. Lin, Brittany Sandoval, Vanessa A. Quevedo Barrios, Veronica Kovalcik, Jonas Koeppel, Elizabeth K. Henderson, Emma C. Fink, Lu Yang, Anthony Chan, Sheela Pangeni Pokharel, Erik J. Bergstrom, Rajan Burt, Namrata D. Udeshi, Steven A. Carr, Eric S. Fischer, Chun-Wei Chen, Benjamin L. Ebert

**Affiliations:** 1 Department of Haematology, UCL Cancer Institute, University College London, London, United Kingdom.; 2 Department of Medical Oncology, Dana-Farber Cancer Institute, Boston, Massachusetts, USA.; 3 Broad Institute of MIT and Harvard, Cambridge, Massachusetts, USA.; 4 Division of Hematology, Brigham and Women’s Hospital, Boston, Massachusetts, USA.; 5 Department of Cancer Biology, Dana-Farber Cancer Institute, Boston, Massachusetts, USA.; 6 Department of Biological Chemistry and Molecular Pharmacology, Harvard Medical School, Boston, Massachusetts, USA.; 7 Department of Medical Oncology and Hematology, University Hospital Zurich and University of Zurich, Zurich, Switzerland.; 8 Division of Cardiovascular Medicine, Department of Medicine, Brigham and Women’s Hospital, Boston, Massachusetts, USA.; 9 Department of Systems Biology, City of Hope Comprehensive Cancer Center, Duarte, California, USA.; 10 Howard Hughes Medical Institute, Boston, Massachusetts, USA.

**Keywords:** Hematology, Therapeutics, Leukemias, Mouse models, Ubiquitin-proteosome system

## Abstract

Targeted protein degradation is a rapidly advancing and expanding therapeutic approach. Drugs that degrade GSPT1 via the CRL4^CRBN^ ubiquitin ligase are a new class of cancer therapy in active clinical development with evidence of activity against acute myeloid leukemia in early-phase trials. However, other than activation of the integrated stress response, the downstream effects of GSPT1 degradation leading to cell death are largely undefined, and no murine models are available to study these agents. We identified the domains of GSPT1 essential for cell survival and show that GSPT1 degradation leads to impaired translation termination, activation of the integrated stress response pathway, and *TP53*-independent cell death. CRISPR/Cas9 screens implicated decreased translation initiation as protective following GSPT1 degradation, suggesting that cells with higher levels of translation are more susceptible to the effects of GSPT1 degradation. We defined 2 Crbn amino acids that prevent Gspt1 degradation in mice, generated a knockin mouse with alteration of these residues, and demonstrated the efficacy of GSPT1-degrading drugs in vivo with relative sparing of numbers and function of long-term hematopoietic stem cells. Our results provide a mechanistic basis for the use of GSPT1 degraders for the treatment of cancer, including *TP53*-mutant acute myeloid leukemia.

## Graphical abstract

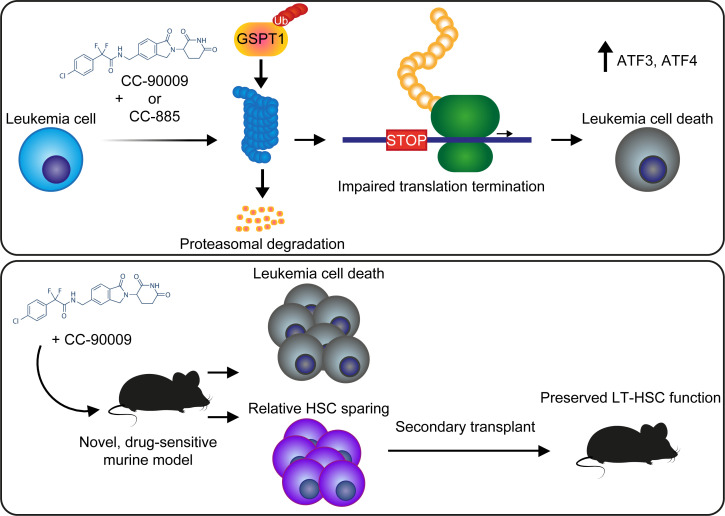

## Introduction

The recent discovery of thalidomide analogs that degrade G_1_ to S phase transition 1 (GSPT1) via the CRL4^CRBN^ ubiquitin ligase highlighted GSPT1 as a novel potential therapeutic target in the treatment of cancer (1). The first-in-class GSPT1 degrader CC-885 demonstrated cytotoxicity across multiple cell lines derived from different tumor types, as well as efficacy against primary samples from patients with acute myeloid leukemia (AML) with relative sparing of lymphocytes. Results from a phase I trial of CC-90009, an analog of CC-885 that also induces degradation of GSPT1, in patients with relapsed/refractory AML has demonstrated promising efficacy (2). More recently, activity of CC-90009 against leukemia stem cells was demonstrated in a xenograft model (3). While CC-90009 treatment was shown to activate the integrated stress response, other mechanisms of cytotoxicity and downstream effects following rapid degradation of GSPT1 are largely undefined. Complicating further study is the lack of murine models that would enable more detailed preclinical studies of toxicity, efficacy, and resistance in vivo.

*GSPT1* is an essential gene for cell survival, and its protein product interacts with multiple factors involved in key cellular processes including regulation of cell cycle progression (4). Through its C-terminus, GSPT1 interacts with ETF1 to form part of the translation termination complex. ETF1 has a transfer RNA–like structure and recognizes the termination codon in the A site of the translating ribosome (5–7). GSPT1 has 4 GTPase domains that, when GSPT1 binds to ETF1, hydrolyze and terminate translation of the nascent protein (8). Knockdown of GSPT1 resulted in increased translation readthrough as well as depletion of ETF1, thought to be related to destabilization of unbound ETF1 (4).

The GSPT1 N-terminal domain contains 2 overlapping PAM2 motifs that specifically recognize the MLLE domain of poly(A)-binding protein (PABP) or UPF1 to coordinate nonsense-mediated decay (NMD) (9, 10). When a termination complex encounters an appropriate stop codon near a poly(A) tail, PABP is brought into proximity with, and binds to, GSPT1. However, if the translation termination machinery encounters a premature stop codon distant from the poly(A) tail and PABP, GSPT1 is instead able to interact with UPF1 and the truncated protein is targeted for NMD (11). The N-terminus also contains a polymorphic polyglycine repeat ranging from 7 to 12 glycines. Population studies suggest that the 12-glycine polymorphism confers a 20-fold increased risk for developing gastric cancer and a 13-fold risk for breast cancer (12, 13). Although the mechanism for such an association is unclear, and the region of GSPT1 reported to bind to PABP is downstream of the polyglycine repeat, a recent study reports that the glycine repeat influences the interaction of GSPT1 with PABP and furthermore that the 12-glycine polymorphism has a 10-fold lower binding affinity for PABP than other GSPT1 isoforms (14). The N-terminus of GSPT1 has also been shown to interact with the antiapoptotic protein survivin (15, 16) and the tumor suppressor p14^ARF^ (17, 18), but with a less clearly defined role in these contexts.

We sought to identify the mechanisms by which rapid degradation of GSPT1 leads to cytotoxicity and the effects on normal hematopoiesis. A challenge in the study of GSPT1 degraders, and thalidomide analogs in general, is the absence of suitable mouse models. Lack of conservation in key amino acids in human CRBN causes many animals, including mice, to be intrinsically incapable of responding to these drugs. In mice, a single amino acid substitution in Crbn^I391V^ is sufficient to confer lenalidomide sensitivity (19). In contrast, CC-885 requires Crbn^V380E^ for GSPT1 degradation (1). The optimal CRBN binding surface for substrate degradation by the clinical-grade compound CC-90009 has not been described. Therefore, we aimed to create a humanized mouse Crbn for the study of GSPT1 degraders and develop an in vivo model sensitive to both CC-885 and CC-90009 to study the efficacy and toxicity of these molecules for the treatment of leukemia.

## Results

### The GSPT1 GTPase and ETF1 binding domains are critical for leukemia cell survival.

To understand the mechanistic basis for GSPT1 dependency in leukemia cells, we first sought to define the domains within GSPT1 critical for leukemia cell survival using an unbiased tiled CRISPR depletion screen. While frameshift deletions anywhere in the *GSPT1* coding region are predicted to lead to complete loss of function, small in-frame deletions are likely to do so only if they impair an essential functional domain. Thus, differential depletion of guides across the length of the gene can be used to assess the importance of functional domains (20). We constructed an sgRNA library including all target sequences with Cas9 PAM sequences (NGG) within the coding sequence of GSPT1 (*n* = 260 sgRNAs). As controls, we included 22 sgRNAs targeting common essential genes, and 41 sgRNAs targeting nonessential sequences (Supplemental Figure 1A; supplemental material available online with this article; https://doi.org/10.1172/JCI153514DS1). Following quality control (QC) assessment of the sgRNA plasmid library by next-generation sequencing, 238 sgRNAs targeting *GSPT1* had representation of more than 5% of expected frequency, giving an average targeting density of 1 sgRNA every 8 bp across the *GSPT1* coding region. The guide sequences and QC results for this library are provided in Supplemental Table 1.

**Figure 1:**
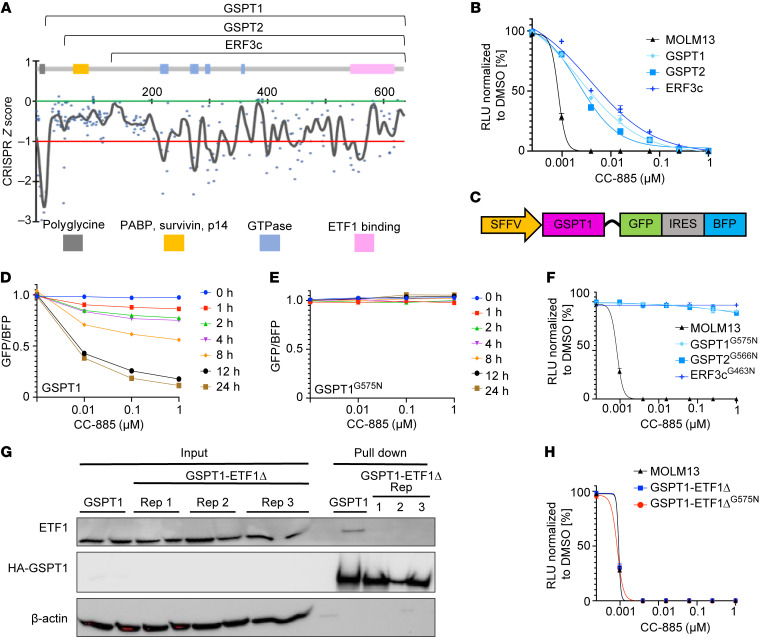
The interaction of GSPT1 with ETF1 is critical for leukemia cell survival. (**A**) Combined CRISPR scores in MOLM13 cells comparing sgRNA representation from triplicates at day 22 versus day 0. Gray, GC-rich (92%) polyglycine repeat; yellow, region containing regions reported to interact with survivin, p14, and poly(A)-binding protein (PABP) involved in nonsense-mediated decay; blue, GTPase domains; pink, ETF1 binding region. Numbers on the *x* axis represent amino acid position. The *y* axis represents CRISPR *z* score and is defined by the median change (day 22 v day 0) in the representation of the negative control sgRNAs (green line = 0.0) and the median change (day 22 v day 0) in the representation of the positive control sgRNAs (red line = –1.0). (**B**) Cell viability of MOLM13 cells expressing GSPT1, GSPT2, and ERF3c constructs. Cell viability was assessed using CellTiter-Glo luminescent assay. (**C**) Design of GSPT1 degradation reporter. (**D** and **E**) Comparison of degradation of GSPT1 (**D**) and GSPT1^G575N^ (**E**). GFP/BFP ratio is relative to DMSO. Representative data of 3 biological replicates. Mean ± SEM of 3 technical replicates. (**F**) Cell viability of MOLM13 cells expressing drug-resistant GSPT1^G575N^, GSPT2^G566N^, and ERF3c^G463N^ constructs. Cell viability was assessed using CellTiter-Glo luminescent assay. (**G**) Immunoprecipitation of HA-GSPT1 and HA-GSPT1 ETF1 binding mutant (ETF1Δ) with mutations in GSPT1 at S541A, I542A, Y547A, F583A, and D618A. (**H**) Cell viability in MOLM13 cells expressing GSPT1-ETF1Δ (drug sensitive and drug resistant; G575N). For all CellTiter-Glo luminescent assays, cell viability was assessed 72 hours after treatment with CC-885 (graphs represent combined data from 3 biological replicates performed in technical triplicate; symbols represent mean ± SEM).

To identify essential domains in GSPT1, we transduced MOLM13 cells with lentivirus expressing the sgRNA library and assessed sgRNA representation from cells harvested at 0 and 22 days after infection by next-generation sequencing (Figure 1A, Supplemental Figure 1, A–D, and Supplemental Table 1). The largest depletion (“dropout”) of sgRNAs was of those targeting the N-terminal region containing a polyglycine repeat, which is 92% GC rich and where potential off-target Cas9 activity is expected. The sgRNAs targeting regions reported to be important for the interaction with survivin and p14 and the region reported to bind PABP showed no loss of representation, suggesting that these regions are not critical for leukemia cell survival and are unlikely to be important for mediating the activity of drug-induced GSPT1 degradation. We observed consistent sgRNA dropout of sgRNAs targeting the GTPase domains and the region implicated in ETF1 binding. Thus, we conclude that ETF1 binding and associated GTPase activity are likely to be the key mediators of leukemia cell death following loss of GSPT1.

### Disruption of ETF1 binding is critical for CC-885–induced cytotoxicity.

We next sought to validate the domains critical for GSPT1 drug-induced degradation and consequent cytotoxicity. The binding of GSPT1 to ETF1, and the subsequent GTPase activity, are key events during translation termination. We examined whether, upon GSPT1 degradation, it is the loss of ETF1 interaction that leads to cytotoxicity. We generated overexpression vectors of WT GSPT1, GSPT2 (a related protein with 97% amino acid homology to GSPT1 but lacking the N-terminus polyglycine GC-rich repeat), and ERF3c (a short isoform of GSPT1 lacking the first 137 amino acids but with an identical C-terminus that includes the GTPase and ETF1 binding domains) (Figure 1A). Overexpression of any of the WT constructs rescued the cytotoxic effects of CC-885 treatment in MOLM13 cells (Figure 1B) and K562 cells (Supplemental Figure 1E).

We sought to rescue cytotoxicity more completely by mutating a glycine (G575) in GSPT1 that was reported to be critical for CC-885–induced degradation (1). We used a fluorescent reporter with either GSPT1 or 1 of 2 mutations at G575 (GSPT1^G575A^ and GSPT1^G575N^) fused to GFP (Figure 1C). The GSPT1^G575A^ mutant partially blocked GSPT1 degradation following CC-885 treatment, at approximately 50% of the WT level of degradation (Supplemental Figure 1, F and G). In contrast, the more structurally disruptive change, GSPT1^G575N^, fully abrogated GSPT1 degradation in this quantitative reporter system (Figure 1, D and E, and Supplemental Figure 1G).

Overexpression of GSPT1^G575N^, GSPT2^G566N^, or ERF3c^G436N^ in MOLM13 cells fully rescued the effects of CC-885 treatment on cell viability (Figure 1F). These findings were replicated in a second AML cell line, K562 (Supplemental Figure 1H). Our results support the hypothesis that the C-terminus of GSPT1, including the ETF1 binding and GTPase domains, is central to the cytotoxic effects of CC-885 treatment. Further supporting this finding, sensitivity of cell lines to loss of GSPT1 is one of the strongest correlations with sensitivity to loss of ETF1 (Broad Institute Dependency Map [DepMap] portal, CRISPR; Avana public library 20Q1; Supplemental Figure 1I). As further validation, we found that CRISPR/Cas9–mediated deletion of *ETF1* sensitized K562 cells to CC-885, suggesting convergence on a common pathway important for mediation of the cytotoxic effect (Supplemental Figure 1J).

To examine the role of the GSPT1-ETF1 interaction in CC-885 activity, we generated GSPT1 constructs that are unable to bind ETF1 and assessed their ability to rescue treatment with CC-885. Using sequence homology with the *Schizosaccharomyces pombe* *Sup35* gene, we generated a GSPT1 construct with 5 amino acid substitutions predicted to disrupt the ETF1 binding interface (S541A, I542A, Y547A, F583A, D618A) (21). Immunoprecipitation of the GSPT1 ETF1 binding domain mutant (GSPT1-ETF1Δ) showed no detectable interaction with ETF1 (Figure 1G) and failed to rescue MOLM13 and K562 viability upon treatment with CC-885 (Figure 1H and Supplemental Figure 1, K and L).

In aggregate, these results demonstrate that, despite its putative role in multiple key cellular processes, the GSPT1 interaction with ETF1, and therefore the role of GSPT1 in translation termination, are key to the cytotoxic effects seen when GSPT1 is acutely degraded.

### CC-885–induced GSPT1 degradation impairs translation termination.

**Figure 2:**
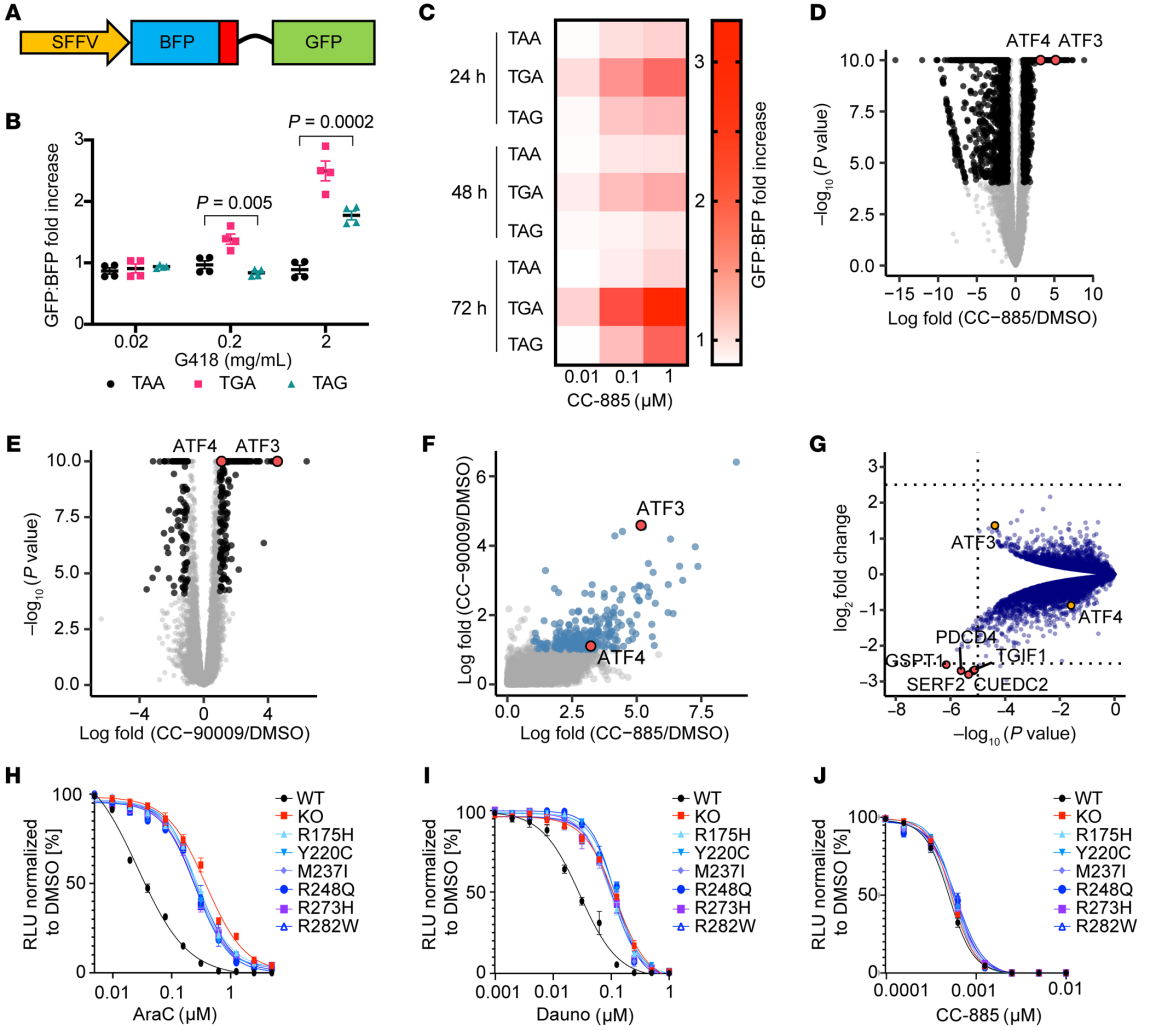
CC-885 treatment impairs translation termination, activates the internal stress response pathway, and results in *TP53-*independent cell death. (**A**) Design of fluorescent translation termination reporter (position of the stop codon is indicated in red). (**B**) GFP/BFP ratio in K562 cells expressing reporters with BFP stop codons following 72 hours of exposure to G418 (ratio expressed relative to DMSO). Results are combined from 4 independent replicates performed in triplicate (Kruskal-Wallis test). (**C**) GFP/BFP ratio in K562 cells expressing reporters with BFP stop codons following 72 hours of exposure to CC-885 (ratio expressed as fold change, indicated by depth of red color, relative to DMSO). Results are combined from 4 independent replicates performed in triplicate. Statistical analysis using Wilcoxon’s test is given in Supplemental Table 2. (**D** and **E**) RNA-Seq results in MOLM13 cells following 6 hours of treatment with 1 μM CC-885 (**D**) and 1 μM CC-90009 (**E**). (**F**) Correlation of gene expression changes in MOLM13 cells following treatment with CC-885 or CC-90009. (**G**) Volcano plot of proteomics data comparing native HEK293T cells with cells overexpressing GSPT1^G575N^ following 18 hours of treatment with 1 μM CC-885. Protein abundance is represented by tandem mass tag (TMT) reporter ion intensity ratios relative to average of DMSO-treated native 293T samples, all measured in duplicate. Significance of a 2-sample modT test between each condition is shown on the *y* axis. Relative change in abundance of proteins between conditions is expressed as fold change on the *x* axis. (**H**–**J**) Cell viability of MOLM13 cells that are *TP53* WT or *TP53* knockout or have endogenous expression of mutant *TP53* following treatment with cytarabine (AraC) (**H**), daunorubicin (Dauno) (**I**), or CC-885 (**J**). CellTiter-Glo luminescent assay 72 hours after treatment; relative luminescent units (RLU) relative to DMSO (graphs represent combined data from 3 biological replicates performed in technical triplicate; symbols represent mean ± SEM).

Having shown that the loss of interaction with the translation termination factor ETF1 is critical to cytotoxicity following GSPT1 degradation, we sought to assess whether GSPT1 loss following CC-885 treatment leads to impaired translation termination. We generated double-fluorescent reporter constructs with BFP linked in-frame to GFP (Figure 2A). At the C-terminus of BFP we introduced 1 of 3 stop codons (TAA, TAG, or TGA) so that GFP would only be expressed following BFP stop codon “readthrough.” Efficacy of the translation termination reporters was verified in K562 cells using the aminoglycoside G418, a drug known to impair translation termination. Treatment with G418 showed a time- and dose-dependent increase in the GFP expression (expressed as a fold change in the GFP/BFP mean fluorescence ratio relative to DMSO) as well as the expected hierarchy of stop codon readthrough (TGA>TAG>>TAA) (22, 23) (Figure 2B and Supplemental Figure 2A).

Following CC-885 treatment we observed a time- and dose-dependent increase in GFP expression levels in BFP^+^ cells indicative of impaired translation termination (Figure 2C). Although the same stop codon readthrough hierarchy as seen for G418 was observed, there was demonstrable readthrough of the TAA stop codon with CC-885 that was not seen following aminoglycoside treatment. We assessed the readthrough using a Wilcoxon’s test according to dose of CC-885, stop codon sequence, and time. At a *P* value of <0.05 the TAG stop codon showed significant increased readthrough with dose increase at 72 hours. For the TGA and TAA there were significant differences between the 0.01 μM and 0.1 μM doses but no significant increase with a dose increase of 0.1 μM to 1 μM. The TAA stop codon showed significantly less readthrough at 72 hours compared with TAG to TGA across multiple doses of CC-885. In contrast, at 72 hours, the only significant difference between TAG and TGA was seen when 0.01 μM was assessed. Statistically significant differences were also seen between TGA and TAA at 48 hours at doses of 0.01 μM and 0.1 μM with no significant difference seen at 1 μM. Full results of statistical analysis are provided in Supplemental Table 2. We conclude that loss of GSPT1 following CC-885 treatment leads to impaired translation termination and inappropriate readthrough of stop codons. The level of readthrough is influenced by the dose of drug used and the stop codon sequence.

### CC-885–induced degradation of GSPT1 leads to activation of the integrated stress response pathway.

To investigate the downstream effects of GSPT1 degradation in an unbiased fashion, we performed RNA-Seq analysis in MOLM13 myeloid leukemia cells treated with CC-885. Comparison with DMSO showed significant upregulation of multiple genes, including *ATF3* and *ATF4*, which are critical nodes in the integrated stress response (ISR) pathway (Figure 2D). We also examined cells after treatment with CC-90009, a GSPT1 degrader currently being assessed in a phase I clinical trial (Figure 2E). In general, treatment with CC-90009 led to an overlapping but more modest effect on gene expression compared with CC-885 (Figure 2F). *ATF3* was also significantly upregulated in CC-90009–treated cells. Gene set enrichment analysis further confirmed that activation of the ISR pathway was a shared downstream event following treatment with GSPT1-degrading drugs (Supplemental Figure 2, B and C).

To determine whether the observed effects were due to degradation of GSPT1, we performed quantitative whole-proteome analysis using tandem mass tag (TMT) labeling and high-resolution liquid chromatography–tandem mass spectrometry to compare native cells, and cells overexpressing GSPT1, or the GSPT1^G575N^ drug-resistant GSPT1 mutant. Following treatment with CC-885, GSPT1 was degraded, as expected, in WT cells compared with cells expressing the drug-resistant GSPT1 (Supplemental Figure 2, D and E, and Supplemental Table 3). In comparison with CC-885–treated GSPT1^G575N^-expressing cells, WT cells showed significant upregulation of ATF3 protein, indicating that the increase in ATF3 is dependent on, and a consequence of, GSPT1 degradation (Figure 2G). We validated these findings in K562 cells by immunoblotting (Supplemental Figure 2F). We also demonstrated increased ATF4 expression dependent on phosphorylation of eIF2a following treatment with CC-885 (Supplemental Figure 2G). In summary, drug-induced GSPT1 degradation causes rapid induction of the ISR pathway at the RNA and protein levels, and maintenance of GSPT1 levels rescues this effect.

### CC-885 cytotoxicity is TP53 independent.

GSPT1 degradation and the resulting impaired translation termination and activation of the ISR pathway represent a novel mechanism of inducing leukemia cell death. Leukemias bearing *TP53* mutations are among the most refractory to therapy and are associated with extremely poor outcomes (24). We sought to assess whether GSPT1 degradation might represent a new approach to treating AML harboring mutations in *TP53*. We examined sensitivity to GSPT1 degradation in a panel of isogenic leukemia cell lines with endogenous expression of WT or mutant *TP53* alleles, including the most common *TP53* mutations in high-risk myelodysplasia/AML (25).

In keeping with the expected pattern of response, *TP53-*mutant and *TP53-*null cells had significantly impaired responses to both cytarabine and daunorubicin, the cornerstones of standard induction chemotherapy (Figure 2, H and I). In contrast, no differences were seen between *TP53-*WT and mutant/null cells following treatment with CC-885 (Figure 2J), demonstrating that treatment with CC-885 results in TP53-independent cytotoxicity. It is noteworthy that CC-885 also degrades casein kinase 1α (CK1α), another important myeloid substrate. However, the cellular effects of CK1α depletion are dependent on an intact TP53 pathway (26), implying that this cytotoxic activity in *TP53-*mutant cells is a result of GSPT1 degradation rather than CK1α degradation.

### CRISPR screens reveal the essential cellular machinery for GSPT1 degradation by CC-885 and mechanisms of resistance.

**Figure 3:**
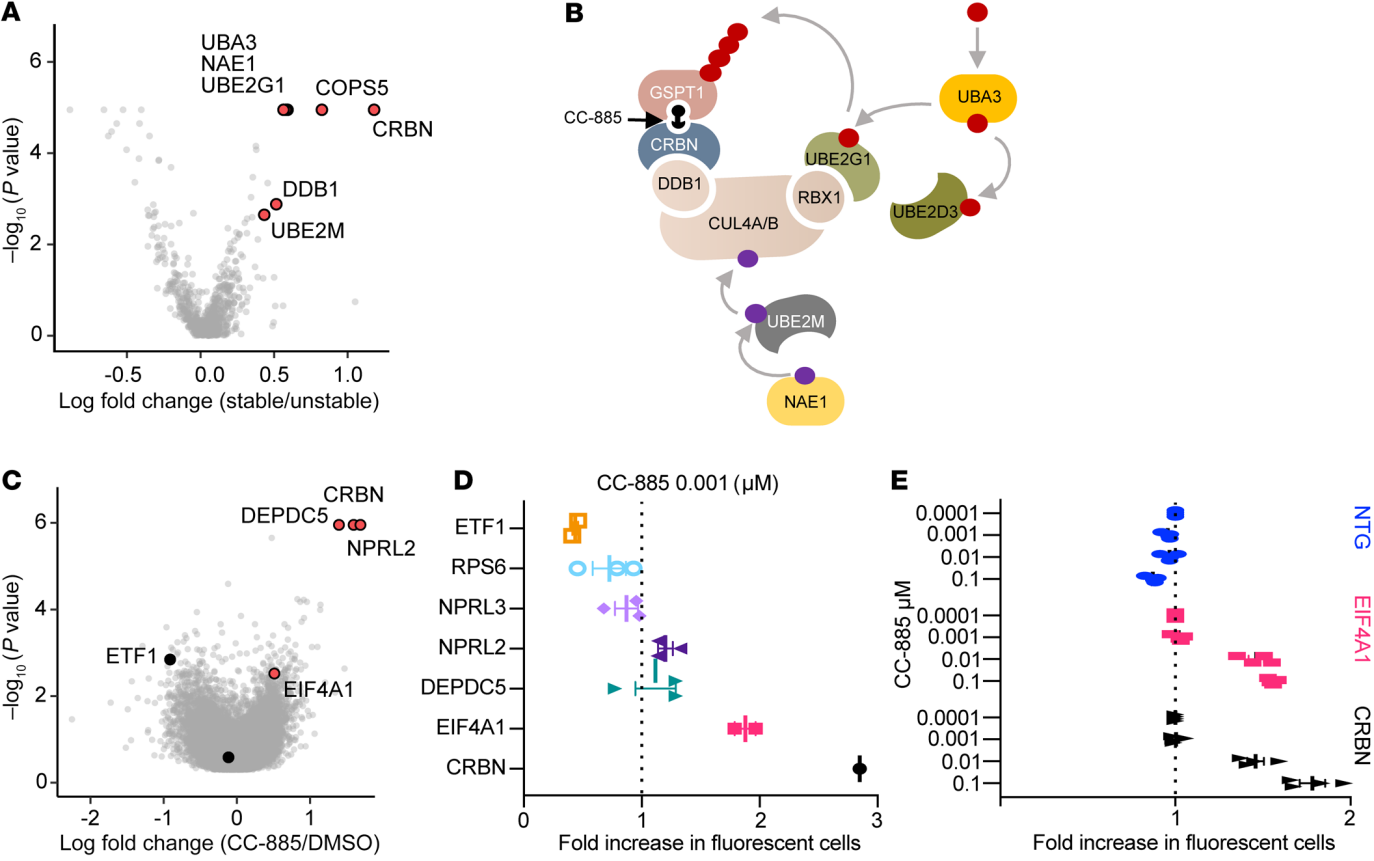
CRISPR screens identify the cullin-RING ligase machinery required for CC-885–dependent CRL4^CRBN^ activity and mechanisms of resistance to CC-885. (**A**) Targeted CRISPR resistance screen showing relative fold enrichment (*x* axis) of sgRNAs in HEK293T cells treated with 1 μM CC-885 relative to DMSO. (**B**) Schematic of molecular machinery required for CC-885–induced degradation of GSPT1. (**C**) Result of genome-wide CRISPR screen showing sgRNAs conferring relative resistance to CC-885 treatment. (**D**) Selected sgRNAs from arrayed validation screen in MM.1S cells showing fold change in representation following 4 days of treatment with 0.001 μM CC-885. (**E**) Fold change in sgRNA representation in K562 cells 4 days after treatment with CC-885 at indicated doses. Data points represent combined change in RFP657^+^ cells for 4 sgRNAs per gene target performed in triplicate ± SEM. NTG, nontargeting guide.

Since *TP53* mutations are a central mechanism of resistance to chemotherapy, and GSPT1 degradation results in TP53-independent cytotoxicity, we next sought to identify mechanisms of resistance to drug-induced GSPT1 degradation. We first performed a targeted CRISPR screen of ubiquitin ligase components (27, 28). As observed in screens for lenalidomide resistance in multiple myeloma, CC-885 requires the components of the CRL4^CRBN^ ubiquitin ligase, genes required for neddylation of cullin-RING ligases, and the UBE2G1 E2 ubiquitin–conjugating enzyme (29) (Figure 3, A and B, and Supplemental Figure 3A).

To identify alternative mechanisms of resistance, we performed a genome-wide CRISPR screen in the *TP53*-WT cell line MM.1S expressing Cas9. As expected, sgRNAs targeting *ETF1* were de-enriched in CC-885–treated compared with DMSO-treated cells (*N* score –2.06, *P* = 0.0014), whereas sgRNAs targeting CRBN were enriched (*N* score 6.8, *P* = 1.11 × 10^–6^). The expected enrichment was also seen for members of the constitutive COP9 signalosome, important for neddylation of cullin 4A (COPS7B and COPS8). Relevant to the role of GSPT1 in translation, we also found sgRNA enrichment in negative regulators of mTORC (*DEPDC5*, *NPRL2*, and *TSC1*) and the key translation initiation factor *EIF4A1* (Figure 3C, Supplemental Figure 3B, and Supplemental Table 4). Importantly, *TP53* did not score in our resistance screen.

To validate these findings, we designed an arrayed sgRNA library focusing on key pathways identified in our genome-wide screen excluding genes identified as part of the core machinery for CRL4^CRBN^ activity, plus sgRNAs targeting *CRBN* and *ETF1* as positive and negative controls (Supplemental Table 5). As expected, using 2 different doses of CC-885, *ETF1* sgRNAs were selectively depleted, and the *CRBN* sgRNA selectively enriched. After *CRBN*, the most robustly enriched sgRNAs (implying relative cell resistance) were those targeting *EIF4A1* (Figure 3D and Supplemental Figure 3C). We next sought to validate loss of EIF4A1 as a resistance mechanism in an additional cell line (K562). Again, sgRNAs targeting *EIF4A1* were selectively enriched following treatment with CC-885 (Figure 3E), suggesting that limiting translation protects cells from CC-885 treatment.

In aggregate our results reinforce the importance of impaired translation termination following drug-induced GSPT1 degradation, as limiting protein translation is relatively protective to cells treated with GSPT1 degraders. As increased translation is a common feature of cancer (30), this provides a potential mechanism whereby normal stem cells would be relatively protected from CC-885 and potentially other drugs targeting GSPT1, providing additional rationale for GSPT1 as a clinical target.

### A double-knockin humanized Crbn mouse (Crbn^V380E/I391V^) is the minimal optimal design for in vivo studies of drug-induced GSPT1 degradation.

We next sought to assess the effects of GSPT1 degradation on normal stem cells, hematopoiesis, and leukemia cells in vivo. However, a critical constraint on the study of thalidomide analog efficacy, toxicity, and resistance is the lack of suitable animal models. While primates are sensitive to thalidomide analogs, mouse cells are intrinsically resistant because of differences in the Crbn amino acid sequence in the drug binding region. Previous studies have demonstrated that humanizing mouse Crbn at I391V is sufficient to induce lenalidomide sensitivity (19), whereas V380E is required for CC-885–induced GSPT1 degradation in vitro (1). However, the optimal changes for degradation of other key substrates of CC-885 such as CK1α are unknown, nor have any potential Crbn changes required for GSPT1 degradation by CC-90009 been described. In addition, mouse and human Crbn also differ at 2 other amino acids, S105F and E152D, at the interface of Crbn, drug, and substrate (Supplemental Figure 4A).

**Figure 4:**
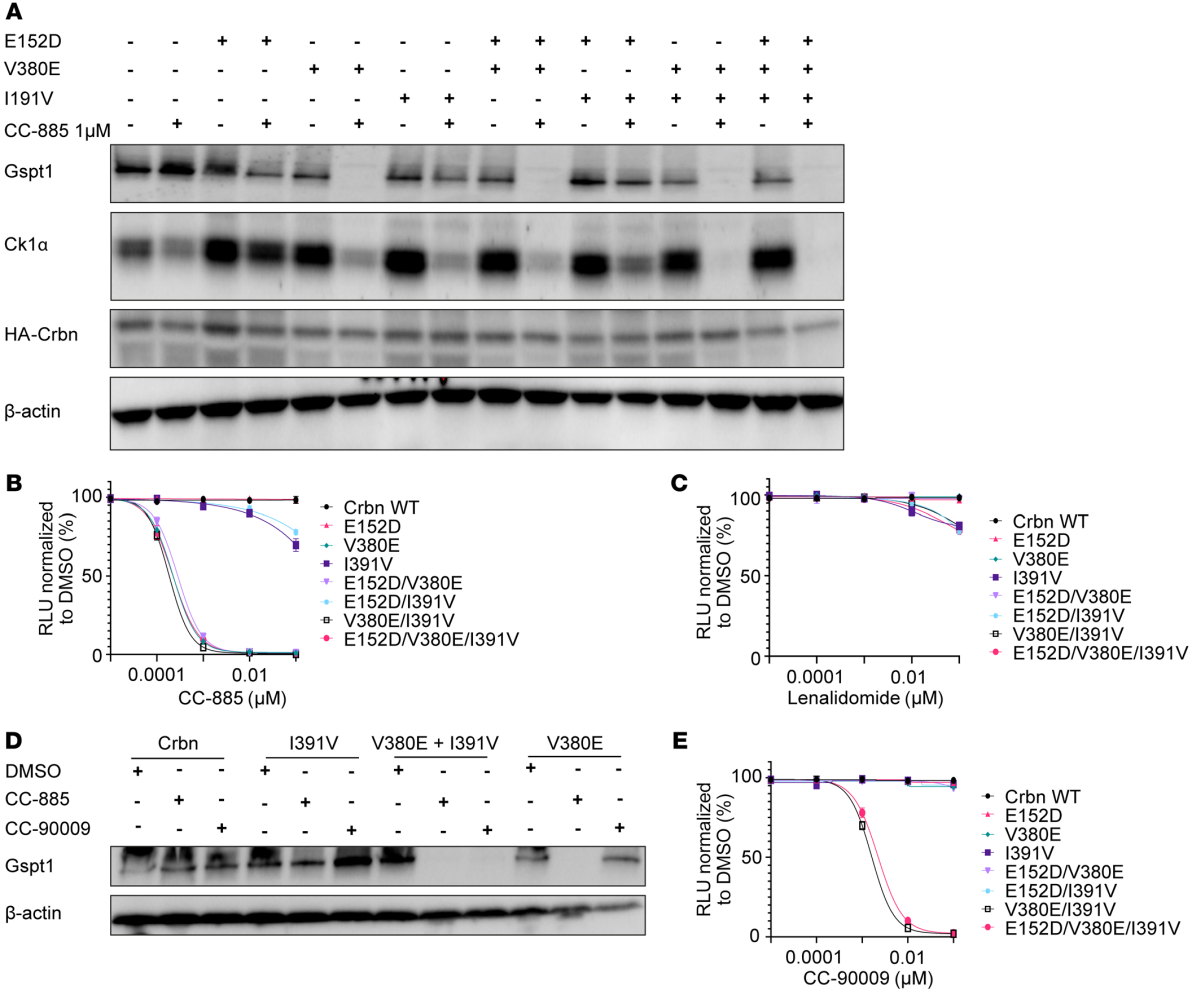
The minimal optimal humanization of mouse Crbn for the investigation of GSPT1-degrading drugs is a double-knockin of V380E and I391V. (**A**) Western blot using lysates from Ba/F3 cells overexpressing Crbn constructs with combinations of humanization mutations E152D, V380E, and I391V as indicated. Protein lysates were harvested following 6 hours of treatment with 1 μM CC-885. (**B** and **C**) Cell viability of Ba/F3 overexpressing Crbn constructs with humanization mutations following treatment with CC-885 (**B**) or lenalidomide (**C**). (**D**) Western blot using cell lysates from Ba/F3 overexpressing Crbn constructs with combinations of humanization mutations as indicated. Protein lysates were harvested following 6 hours of treatment with either CC-885 or CC-90009 at 1 μM. (**E**) Cell viability of Ba/F3 overexpressing Crbn constructs with humanization mutations following treatment with CC-90009. For **B**, **C**, and **E**, cell viability was assessed using CellTiter-Glo luminescent assay 72 hours after treatment (graphs represent combined data from 3 biological replicates performed in technical triplicate; symbols represent mean ± SEM).

We expressed all combinations of *Crbn* S105F, E152D, V380E, and I391V in the mouse leukemia cell line Ba/F3 and assessed the impact on protein degradation and cell viability following exposure to CC-885 (Figure 4, A–C, and Supplemental Figure 4, B–D). We observed degradation of Gspt1 in cells expressing constructs containing V380E, with no impact seen with other humanized changes alone or in combination. These findings correlated with a marked decrease in cell viability at 72 hours in cells expressing constructs containing V380E, with no apparent increased efficacy when combined with additional humanized changes. In contrast, for the substrate CK1α, an important substrate when considering hematopoietic progenitor cells and hematological disease, partial degradation was seen with either the V380E or the I391V mutation. However, more marked degradation was observed in cells expressing constructs containing both humanized changes (Figure 4A and Supplemental Figure 4B). Moreover, analysis of cells expressing Crbn constructs with combinations of V380E and I391V revealed that for CC-90009, a GSPT1-degrading drug in phase I clinical trials, V380E alone is insufficient for efficacious Gspt1 degradation. Crbn containing both V380E and I391V is required for Gspt1 degradation in this context (Figure 4, D and E, and Supplemental Figure 4E). These results support the minimal optimal design of a mouse model sensitive to GSPT1-degrading drugs, including the first clinical compound, as having Crbn humanized at both V380E and I391V.

### Leukemia developed from Crbn^V380E/I391V^ mice is sensitive to drugs that degrade Gspt1.

**Figure 5:**
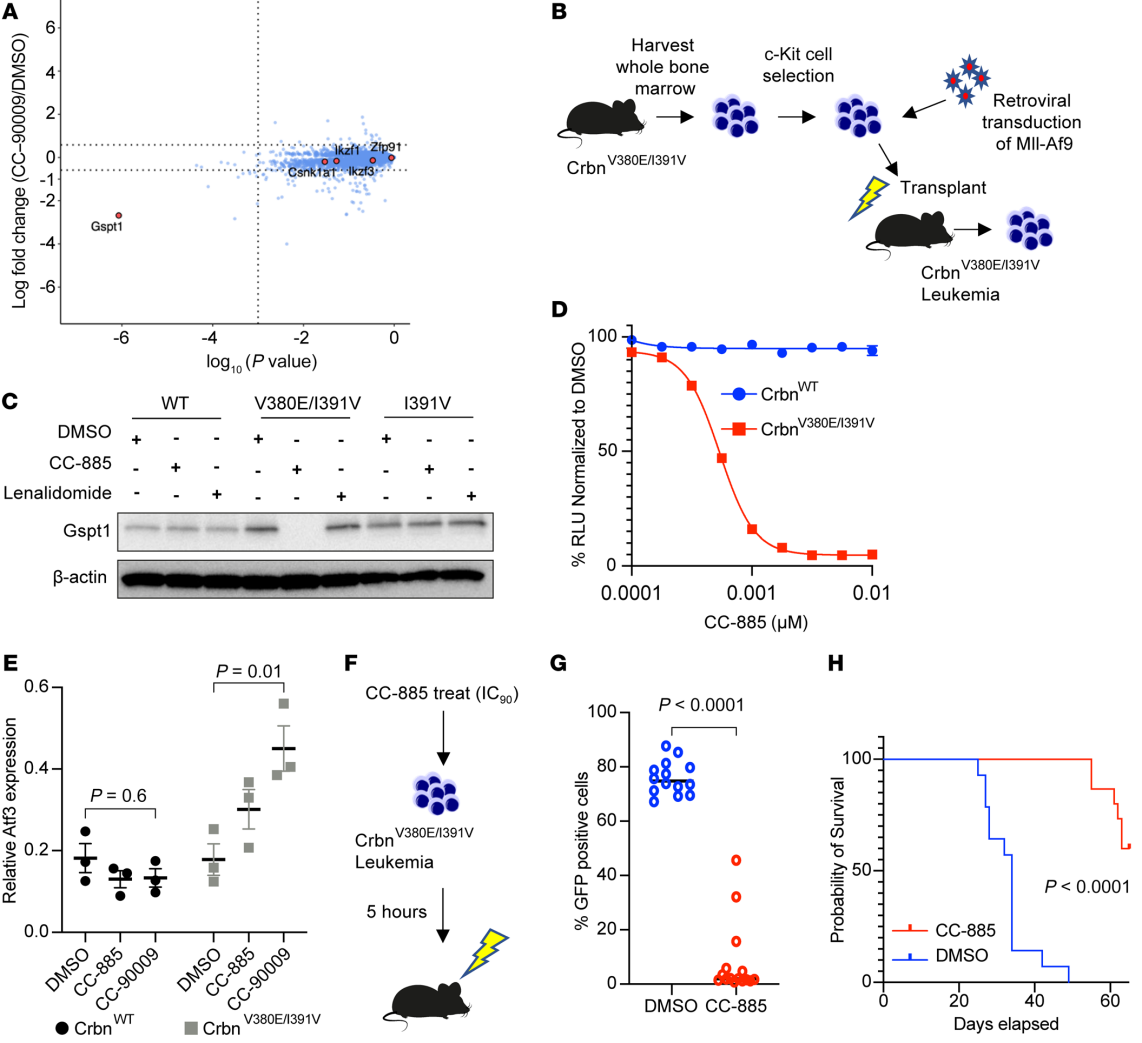
Drugs that degrade Gspt1 are efficacious in leukemia models developed in *Crbn^V380E/I391V^* mice. (**A**) Whole-proteome analysis of c-Kit^+^ cells from *Crbn^V380E/I391V^* mice following treatment with CC-90009. (**B**) Outline for development of primary *Mll-Af9* leukemia. (**C**) Western blot of whole-cell lysates from primary *Mll-Af9* leukemia cells developed in different Crbn backgrounds (WT, V380E/I391V, or I391V) as indicated. Cells were incubated for 6 hours with DMSO, CC-885, or lenalidomide at 1 μM before harvest. (**D**) Cell viability of *Mll-Af9 Crbn^V380E/I391V^* leukemia in different Crbn backgrounds as indicated, measured at 72 hours following treatment with CC-885 using CellTiter-Glo luminescent assay. Relative luminescent units (RLU) relative to DMSO. Plots represent combined data of 3 independent replicates performed in triplicate showing mean ± SEM. (**E**) Reverse transcriptase PCR of *Mll-Af9 Crbn^V380E/I391V^* cells treated with 0.1 μM CC-885 or 1 μM CC-90009 for 24 hours. Combined results of 3 experiments performed with 3 technical replicates (mean ± SEM). *P* values are from Kruskal-Wallis test. (**F**) Design of transplant experiment. Before transplant, *Mll-Af9 Crbn^V380E/I391V^* cells were treated for 5 hours with either DMSO (*n* = 14) or 0.001 μM CC-885 (approximate IC_90_) (*n* = 15). (**G**) GFP expression in peripheral white blood cells 3 weeks after secondary transplants of *Mll-Af9 Crbn^V380E/I391V^* leukemia. (**H**) Survival in days following secondary transplants with *Mll-Af9 Crbn^V380E/I391V^* leukemia according to pretransplant treatment with DMSO (*n* = 14) or 0.001 μM CC-885 (*n* = 15).

Based on our in vitro mutagenesis studies, we generated a knockin mouse model constitutively expressing Crbn with the humanization changes V380E and I391V on the same allele (*Crbn^V380E/I391V^*). The targeting construct to develop the *Crbn^V380E/I391V^* mouse and the confirmatory genotyping of bred homozygous mice are shown in Supplemental Figure 5, A–F. To ensure that introduction of V380E/I391V into Crbn did not interfere with any normal functions of Crbn during mouse hematopoiesis, we performed a detailed characterization of the hematopoietic system in homozygous transgenic animals as compared with WT controls. Relative to WT mice, *Crbn^V380E/I391V^* mice had no differences in peripheral blood or bone marrow counts, lymphocyte subsets in the blood or spleen, or stem or progenitor cell counts in the bone marrow (Supplemental Figure 5, G–N). In addition, treatment of primary c-Kit^+^ cells from the double *Crbn^V380E/I391V^* mouse with pomalidomide, CC-885, or CC-90009 followed by global quantitative mass spectrometry–based proteomics demonstrated degradation of known drug targets including Gspt1, whereas the Crbn^I391V^ demonstrated degradation of known targets only in the presence of pomalidomide (Figure 5A and Supplemental Figure 5O). Full proteomic results of these experiments are provided in Supplemental Table 6.

To generate a murine leukemia that is sensitive to thalidomide analogs, we transduced c-Kit^+^ cells from homozygous *Crbn^V380E/I391V^* mouse bone marrow with retrovirus produced from the pMIG *Mll-Af9* vector. We transplanted transduced cells into sublethally irradiated WT mice and harvested leukemia cells after the development of aggressive disease at 7 weeks (Figure 5B). Treatment of *Mll-Af9* leukemia cells with CC-885 caused effective degradation of Gspt1 (Figure 5C). We performed global whole-proteome analysis on *Mll-Af9* leukemias in *Crbn^WT^*, *Crbn^I391V^*, or *Crbn^V380E/I391V^* background treated with pomalidomide, CC-885, or CC-90009. CC-90009–treated *Crbn^V380E/I391V^* cells showed relatively specific degradation of GSPT1/2 (Supplemental Figure 5P). In contrast, CC-885 also showed degradation of Ck1α (Supplemental Figure 5P), with no effect of either drug seen in the *Crbn^WT^* or *Crbn^I391V^* background. Full quantitative proteomic results of these experiments are provided in Supplemental Table 7. We also demonstrated decreased cell viability (Figure 5D), as well as the expected increase in *Atf3* mRNA on treatment with either CC-885 or CC-90009, thus validating the model (Figure 5E). Comparing *Atf3* mRNA from *Crbn^WT^* versus *Crbn^V380E/I391V^* leukemias after treatment with DMSO, CC-885, and CC-90009 gave *P* values of 0.95, 0.03, and 0.006, respectively. Since CC-885 treatment is highly toxic in vivo, we treated derived leukemia cells for 5 hours with DMSO or 0.001 μM CC-885 before secondary transplantation of 10,000 viable cells into sublethally irradiated WT mice. Mice receiving CC-885–treated cells had a significantly decreased disease burden compared with mice receiving DMSO-treated cells (Figure 5, F and G), and a significant increase in survival (*P* < 0.0001; Figure 5H). These results demonstrate that the *Crbn^V380E/I391V^* mouse does not have perturbed hematopoiesis at baseline and degrades Gspt1 and Gspt2 in response to CC-885/CC-90009 treatment.

### A Crbn^V380E/I391V^ double-knockin mouse is sensitive to in vivo drug-induced Gspt1 degradation.

Our *Crbn^V380E/I391V^* murine model provides an opportunity to characterize the effects of Gspt1 degradation on normal hematopoiesis. We assessed the relative impact of in vivo treatment with the GSPT1 degrader CC-90009 on *Crbn^V380E/I391V^* versus WT cells. To compare the effect of treatment on hematopoietic cells within the same animal, we transplanted *Crbn^V380E/I391V^* (CD45.2) and *Crbn^WT^* (CD45.1) c-Kit–selected cells into lethally irradiated recipient mice at a 50:50 ratio (Supplemental Figure 6A). Following recovery and full hematopoietic reconstitution, we treated the mice with CC-90009.

At a dose of 2 mg/kg CC-90009 once daily by oral gavage for 5 days, we observed no significant effect on peripheral blood chimerism (Supplemental Figure 6B). At a dose of 20 mg/kg once daily, we observed a rapid reduction in CD45.2 chimerism, with the most pronounced effect on B220^+^ B cells, followed by CD11b^+^ myeloid cells (Supplemental Figure 6, C–E). At the end of treatment, we observed a plateauing of the CD45.2 composition, suggesting preservation of CD45.2 stem cells upon cessation of treatment.

**Figure 6:**
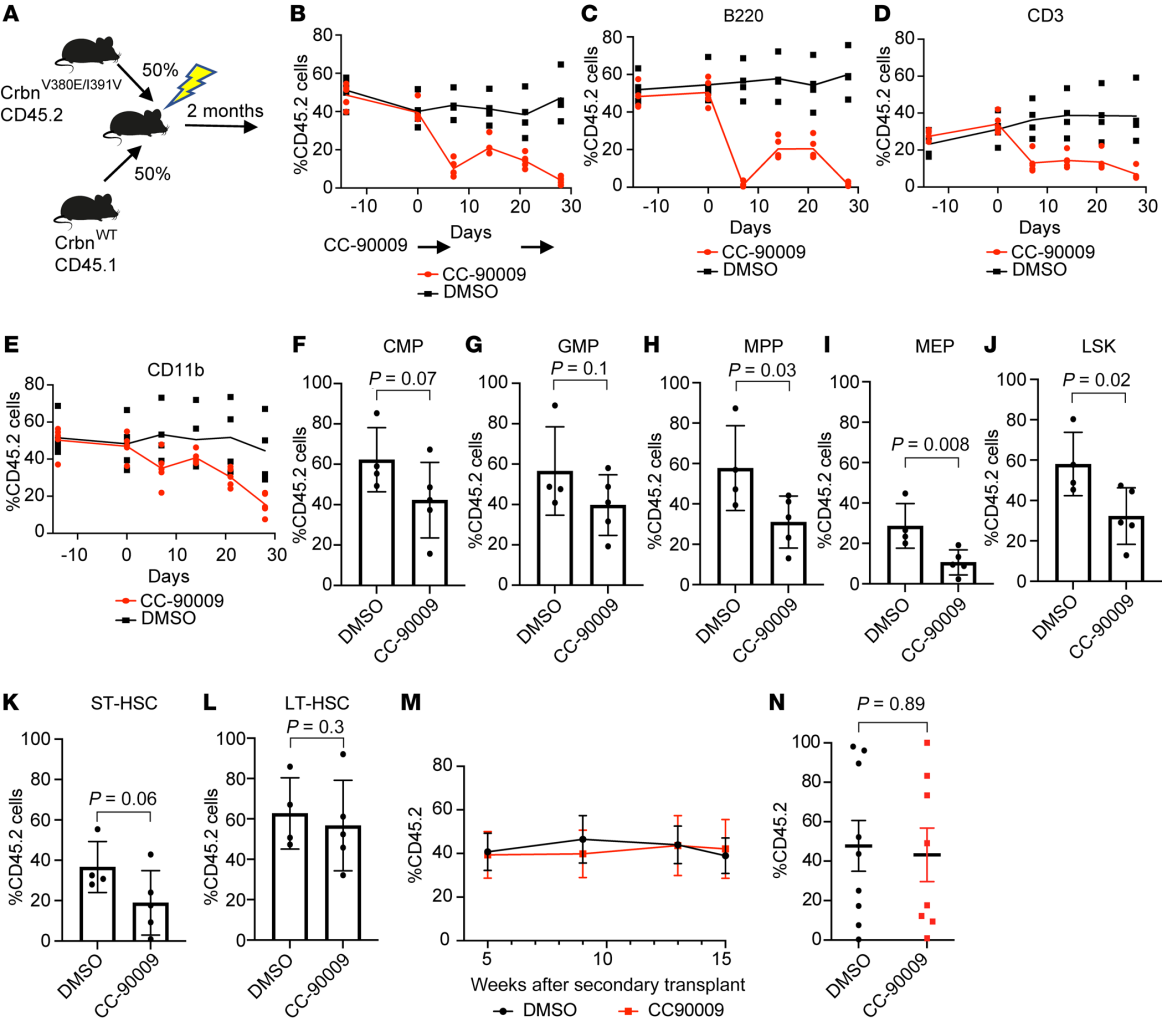
In vivo treatment with CC-90009 selectively targets *Crbn^V380E/I391V^* cells. (**A**) Design of mixed-chimera mice with transplants of c-Kit–selected cells, 50% *Crbn^V380E/I391V^* (CD45.2), and 50% *Crbn^WT^* (CD45.1) following irradiation of recipient animals. (**B**) Peripheral blood chimerism based on percentage CD45.2 cells. Mice were treated with CC-90009 by oral gavage once daily at a dose of 20 mg/kg from day 0 to day 4 and from day 20 to day 24. (**C**–**E**) Peripheral blood chimerism based on percentage CD45.2 cells in B cells (CD220^+^) (**C**), T cells (CD3^+^) (**D**), and myeloid cells (CD11b^+^) (**E**) in the same transplant/treatment schedule. (**F**–**L**) Bone marrow chimerism according to percentage CD45.2 cells in each stem cell compartment. (**F**) Common myeloid progenitor (CMP: Lineage^lo^, c-Kit^+^, Sca1^–^, CD34^+^CD16/32^lo^). (**G**) Granulocyte myeloid progenitor (GMP: Lineage^lo^, c-Kit^+^, Sca1^–^, CD34^+^CD16/32^hi^). (**H**) Multipotent progenitor (MPP: LSK, CD48^+^CD150^–^). (**I**) Megakaryocyte erythroid progenitor (MEP: Lineage^lo^, c-Kit^+^, Sca1^–^, CD34^–^CD16/32^lo^). (**J**) LSK (Lineage^–^, c-Kit^+^, Sca1^+^). (**K**) Short-term hematopoietic stem cell (ST-HSC: LSK, CD48^–^CD150^–^). (**L**) Long-term hematopoietic stem cell (LT-HSC: LSK, CD48^–^CD150^+^). Differences between groups (*n* = 5 per group) were assessed using 1-tailed unpaired *t* test. All differences were statistically nonsignificant at a cutoff of *P* = 0.05. (**M**) Chimerism of CD11b^+^ cells from peripheral blood taken at indicated times after secondary transplant. (**N**) Chimerism in LT-HSC compartment assessed 15 weeks after secondary transplant. DMSO and CC-90009 indicate in vivo treatment given to donor mice. Mice per group: DMSO, *n* = 9; CC-90009, *n* = 8.

We repeated this experiment using 2 cycles of 20 mg/kg dosing (Figure 6, A and B). Again, there was a marked decrease in CD45.2 chimerism in peripheral blood with the most marked effect seen in B cells compared with CD11b^+^ myeloid cells (Figure 6, B–E). After completion of the second cycle of CC-90009 treatment, bone marrow was harvested and CD45.2 chimerism assessed in different stem cell compartments (Figure 6, F–L). Notably, and of potential relevance to clinical application of GSPT1 degraders, there was minimal impact on the chimerism in the long-term hematopoietic stem cell (LT-HSC) compartment, suggesting that long-term repopulating stem cells may be spared (Figure 6L).

To assess the functional output of these LT-HSCs, we used harvested bone marrow from these mice to perform secondary transplants into lethally irradiated CD45.1 WT recipients and assessed chimerism in CD11b^+^ myeloid cells in peripheral blood at 5, 9, 13, and 15 weeks after transplant (Figure 6M). There was no difference in contribution to CD11b^+^ cells between mice previously treated with CC-90009 versus vehicle control. In keeping with this, when bone marrow was assessed at 15 weeks after transplant, there was no difference between prior treatment groups in chimerism assessed in the LT-HSC compartment (DMSO mean = 41 vs. CC-90009 mean = 39, *P* = 0.89; Figure 6N). Other stem and progenitor cell compartments were similarly unaffected (Supplemental Figure 6, F–J).

**Figure 7:**
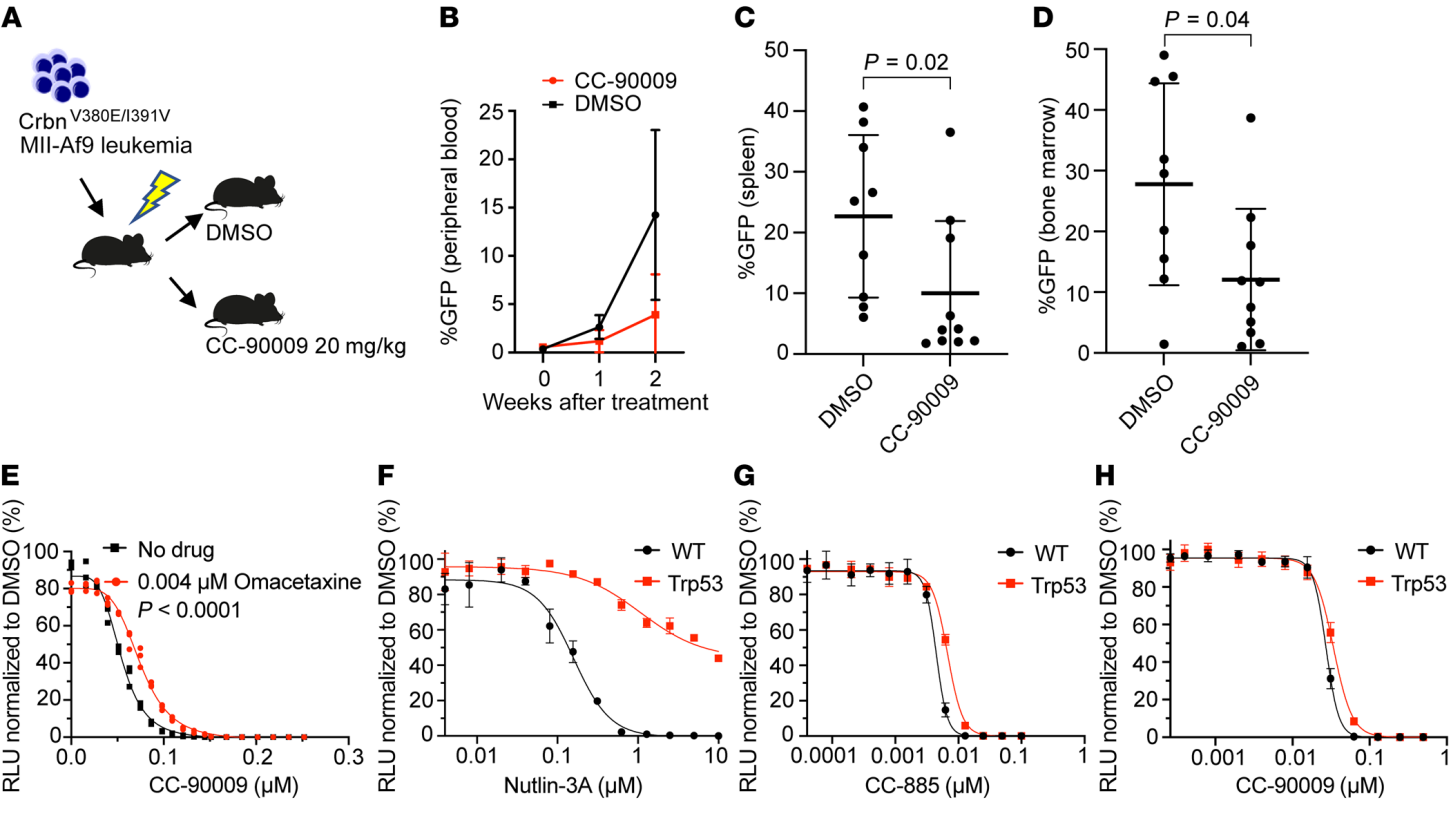
In vivo treatment with CC-90009 can treat *Crbn^V380E/I391V^* leukemia. (**A**) Design of secondary transplant experiment. (**B**) Assessment of GFP^+^ (*Mll-Af9*) cells in peripheral blood in DMSO-treated and CC-90009–treated (20 mg/kg) mice before treatment and at 1 week and 2 weeks after treatment. (**C** and **D**) Assessment of disease burden (GFP^+^ cells) in spleen (**C**) and bone marrow (**D**) 2 weeks after treatment with either DMSO or CC-90009 (20 mg/kg). (**E**) Cell viability in *Mll-Af9 Crbn^V380E/I391V^* cells treated with 0.004 mM omacetaxine in combination with CC-90009 at the indicated doses. Cell viability was assessed using CellTiter-Glo luminescent assay 72 hours after treatment (shown is a representative experiment performed in technical triplicate). (**F**–**H**) Cell viability in *Mll-Af9 Crbn^V380E/I391V^* cells in a *TP53-*WT or *TP53-*null (*Trp53*) background as indicated following treatment with nutlin (**F**), CC-885 (**G**), and CC-90009 (**H**). Cell viability was assessed using CellTiter-Glo luminescent assay 72 hours after treatment (shown are representative experiments performed in technical triplicate).

We then assessed the efficacy of CC-90009 to treat leukemia in vivo. After sublethal irradiation, 10,000 viable *Crbn^V380E/I391V^* *Mll-Af9* cells were transplanted into *Crbn^WT^* mice (Figure 7A). Mice were bled weekly to check for evidence of GFP^+^ cells in the peripheral blood. After the initial appearance of GFP^+^ cells, mice were treated with 20 mg/kg CC-90009 or vehicle control given once daily by oral gavage for 12 days. Treated mice had a significant reduction in leukemic disease burden in all hematopoietic organs (peripheral blood, bone marrow, and spleen) (Figure 7, B–D).

These data demonstrate that murine cells expressing *Crbn^V380E/I391V^* are uniquely capable of responding to GSPT1-degrading drugs such as CC-90009 and establish this, to our knowledge, as a new mouse model in which to study the toxicity and activity of these agents. Furthermore, these data demonstrate that CC-90009 is active in a *Crbn^V380E/I391V^* in vivo mouse model of leukemia.

To further assess whether CC-90009 toxicity was mediated by aberrant translation termination in this leukemia model, we treated cells with omacetaxine, an inhibitor of protein translation that is approved for the treatment of chronic myeloid leukemia (31). Cells treated with omacetaxine were relatively spared from the cytotoxic effects of CC-90009, suggesting that inhibition of translational elongation can partially compensate for the impaired translation termination seen following Gspt1 degradation (Figure 7E). A comparison of best-fit curves using nonlinear regression showed a statistically significant protective effect (IC_50_ of 0.054 μM vs. 0.075 μM, no omacetaxine vs. omacetaxine, *P* < 0.0001). We then generated *TP53-*null *Mll-Af9* leukemia from c-Kit^+^ cells harvested from *Crbn^V380E/I391V^* mice crossed with *Trp53* mice (*Tp53* null). The *Tp53-*null leukemia showed the expected limited response to nutlin-3A, an inhibitor of MDM2-mediated proteasomal degradation of p53, when compared with *Tp53*-WT *Mll-Af9* leukemia (Figure 7F). In contrast, responses to CC-885 and CC-90009 were similar between *Tp53-*WT and -null leukemia, supporting a p53-independent mechanism of cell death (Figure 7, G and H).

## Discussion

Thalidomide analogs that degrade GSPT1 are a new class of drug that show activity against a broad range of cell lines and have recently entered early-phase clinical trials in relapsed/refractory AML with early signs of efficacy (1, 2, 32). We sought to examine the mechanistic basis for the activity of these agents and develop new models for their study. We demonstrate that, despite multiple biological roles, it is the interaction of GSPT1 with ETF1 and the resulting GTPase-dependent translation termination that are key to cell survival. We show that when this interaction is lost as a result of GSPT1 degradation, impaired translation termination with stop codon readthrough occurs. In concordance with a prior study, we show that GSPT1 degradation leads to activation of the ISR pathway and cytotoxicity in leukemia cells (3). In addition, we show that this response is seen in leukemia cells harboring mutations in *TP53*. To assess in vivo efficacy, we developed what is, to our knowledge, a new mouse model with humanized *Crbn* and demonstrate effective Gspt1 degradation following treatment with 2 different degraders. While *Mll-Af9* leukemias are effectively killed in this model, LT-HSCs are relatively spared, an important finding for the clinical translation of GSPT1-degrading drugs.

Degradation of GSPT1 and the resulting impaired translation termination and activation of the ISR are a new approach to cancer treatment. We investigated whether this might prove efficacious in high-risk AML patients, who have not seen improved survival from the introduction of novel therapies. In contrast to drugs used in standard therapy, treatment of isogenic cell lines with CC-885 killed *TP53-*mutant cell lines with similar efficacy to isogenic *TP53*-WT cells. Therefore, in the context of GSPT1 degradation, it appears that intact TP53 is not a sine qua non for an effective cytotoxic effect. Should this translate into clinical studies, it could be a significant improvement on currently available therapy for patients with the highest-risk disease. The potential for efficacy of CC-90009 in high-risk AML has also recently been suggested in a xenograft model of AML, though that model used transcriptional profiles rather than a clinically defined high-risk molecular subtype (3).

Neither *Crbn^WT^* nor *Crbn^I391V^* mice are able to degrade Gspt1 following treatment with either CC-885 or the clinical compound CC-90009. Therefore, while studies using human xenografts are able to demonstrate antileukemia effects, they cannot inform regarding drug toxicity, including the effects of GSPT1 degraders on normal hematopoiesis. To address this, we designed an in vivo model to study the effects of GSPT1 degraders. We showed that the V380E humanization of mouse Crbn is sufficient for effective in vitro degradation of Gspt1 and cytotoxicity with CC-885; however, humanization changes at both V380E and I391V are required together for effective degradation of the key myeloid substrate Ck1α with CC-885 as well as Gspt1 with CC-90009. Interestingly, this was not predicted from crystal structures of CC-90009 bound to CRBN, where V380E was predicted to be unimportant for GSPT1 degradation; this highlights the importance of integrating structural studies and functional assays.

Having created a *Crbn^V380E/I391V^* mouse model and demonstrated that it has no effect on normal hematopoiesis, we generated *Mll-Af9* leukemias. We demonstrated effective degradation of Gspt1, induction of the ISR pathway, and cytotoxicity on treatment with Gspt1 degraders. In vivo experiments using the clinical compound CC-90009 showed selective sensitivity for *Crbn^V380E/I391V^* cells and a reduction in disease burden in mice with established leukemia. Crucially, we demonstrated that although CC-90009 does affect peripheral blood counts and aspects of normal hematopoiesis, the LT-HSC compartment was minimally affected following treatment with CC-90009, including preserved functional output following secondary transplantation. This is in contrast to the activity of CC-90009 in leukemia stem cells (3) and has potential implications for effective recovery of hematopoiesis following treatment in the clinic. Supporting our observations in the *TP53-*mutated isogenic cell lines, *Crbn^V380E/I391V^ Mll-Af9 Tp53-*null leukemia was effectively killed following treatment with either CC-885 or CC-90009.

Using CRISPR screens and validation studies, we show the importance of impaired translation termination to the cytotoxic effects of GSPT1 degraders. Furthermore, limiting translation through genetic perturbation of EIF4A1 or pharmacological treatment with the translation inhibitor omacetaxine confers relative resistance to GSPT1 degradation. Thus, the relative sparing of LT-HSCs may be related in part to their highly regulated and inherently lower level of protein translation (33–35).

The demonstration of the importance of protein translation to drug efficacy and resistance is of potential importance for clinical studies. A central tenet of combination chemotherapy is the use of agents with orthogonal mechanisms of action and resistance. Our studies demonstrate that the cellular consequences of GSPT1 degradation are entirely different from those of established chemotherapy agents. Furthermore, the observed mechanisms of resistance involving reduced translation are fundamentally different from described mechanisms of drug resistance. Thus, drugs that degrade GSPT1 may be useful additions to combination regimens to increase efficacy or reduce the emergence of resistant disease. They also may provide an essentially different approach to treatment in the setting of relapsed or refractory disease.

In aggregate, our results demonstrate the central importance of translation and impaired translation termination to the cytotoxicity of GSPT1 degraders. We generated a novel double-knockin *Crbn^V380E/I391V^* mouse for the evaluation of efficacy and safety of this class of drugs. Using this model, we were able to demonstrate the efficacy of Gspt1 degraders in a leukemia model while preserving LT-HSCs. Our results provide a mechanistic basis for the use of GSPT1 degraders for the treatment of cancer, including *TP53*-mutant AML.

## Methods

### CRISPR domain screen

#### Preparation of DNA library.

The sgRNA library included all sgRNAs for the Cas9 protospacer adjacent motif (PAM) within the *GSPT1* cDNA sequence (NM_002094.3) and control sgRNAs targeting essential and nonessential genes (positive and negative controls, respectively). Target sequences and genes are provided in Supplemental Table 1. Details of library and virus preparation are provided in Supplemental Methods.

#### Screen.

At 48 hours after viral transduction (day 0), transduction efficiency was assessed by percentage of cells expressing RFP, and an aliquot of cells for each triplicate was harvested and frozen at –20°C. The remaining cells were passaged in puromycin, and further cells frozen with each passage/split. Cell pellets and passaged cell numbers were sufficient to allow a minimum of ×2000 sgRNA representation. Cells were kept in culture until day 22 (to allow at least 10,000-fold expansion).

#### Analysis.

Genomic DNA of the screen cell samples was extracted from the triplicates on days 0, 12, and 22. The incorporated sgRNA sequences were amplified by PCR and sequenced by NextSeq550 (Illumina) (36). The frequency for individual sgRNA was calculated by the read number of each sgRNA divided by the total sequenced reads in this library and analysis using the edgeR package (RRID:SCR_012802) (36, 37). The CRISPR score was defined as the change of frequency (i.e., ratio) of individual sgRNA at the end (day 22) versus at the start (day 0) of the screen. For example, a CRISPR score of 0.1 indicates a 10-fold depletion of the sgRNA frequency in the screen.

#### Screen validation.

Overexpression constructs were created using sequence-verified gBlocks (Integrated DNA Technologies) for GSPT1, GSPT2, ERF3c, and the respective G>N mutants. The ETF1 binding mutant and G>N construct were generated using sequence-verified gBlocks and overlapping primers. These were cloned into a lentiviral vector also expressing GFP and puromycin resistance gene. Lentivirus was made as above with the VSV-G (Addgene 12259) and psPAX2 plasmids (Addgene 12260).

K562 (NCI Developmental Therapeutics Program catalog K-562, RRID:CVCL_0004) and MOLM13 (RRID:CVCL_WI20) cell lines were obtained from the Broad Institute cell line repository and were cultured in RPMI, 100 U/mL penicillin, and 100 mg/mL streptomycin with 10% FBS in a 37°C incubator at 5% carbon dioxide. Cells were spinfected with virus at 800*g* in the presence of 4 μg/mL Polybrene. After 48 hours, cells were selected with puromycin at 2 μg/mL for 96 hours.

For the drug sensitivity assays, 2 × 10^4^ cells were seeded per well in a 96-well flat-bottom plate. CC-885 was added to wells in limiting dilutions (Tecan D300e Digital Dispenser). After 72 hours of drug exposure, cell viability was assessed by CellTiter-Glo luminescent assay (Promega G7572) using a FilterMax F5 (Molecular Devices) plate reader. Cell viabilities were calculated relative to DMSO controls.

### Degradation reporter assay

Artichoke plasmid (Addgene 73320) was used. The mCherry sequence was removed by restriction digest. BFP sequence was amplified by PCR, digested, and ligated into the linearized vector to create a GFP-IRES-BFP reporter. GSPT1, GSPT1^G575A^, and GSPT1^G575N^ sequence-verified gBlocks (Integrated DNA Technologies) were amplified using standard Phusion PCR (Thermo Fisher Scientific) with BsmBI restriction sites introduced by forward and reverse primer sequence. PCR products were purified using the QIAquick PCR Purification Kit (Qiagen), digested with BsmBI (New England Biolabs), and ligated into the reporter vector using the T4 DNA ligase (New England Biolabs).

Virus was created as before and K562 cells transduced and puromycin-selected. Cells were plated in 96-well plates at 2 × 10^4^ cells per well. Live cells based on forward and side scatter were assessed after the time and dose indicated by comparison of the mean fluorescence intensity (MFI) of GFP to BFP in treated cells versus DMSO. Flow cytometry was performed on a FACSCanto II (BD Biosciences).

### Immunoprecipitation

HA-GSPT1 and HA-GSPT1 with mutated ERF1 (ETF1Δ) binding were cloned into lentiviral vector, virus made as above, and HEK293T cells transduced and puromycin-selected for 96 hours (4 μg/mL). Cells were lysed in Pierce IP lysis buffer (Thermo Fisher Scientific 87787) freshly supplemented with Halt Protease and Phosphatase Inhibitor Cocktail (Thermo Fisher Scientific 78440). Part of the lysate was incubated with HA-tagged magnetic beads, and the supernatant was removed in the presence of a magnet and washed with cold PBS 3 times before extraction of the immunoprecipitate. Protein concentration of the input and immunoprecipitate was quantified using a Pierce BCA Protein Assay Kit (Thermo Fisher Scientific 23225). Five micrograms of input and 100 μg of immunoprecipitate were loaded and run on a polyacrylamide gel, transferred to PVDF membranes, and blotted for ETF1, HA, and β-actin (Abcam ab173838). Goat anti-mouse–HRP (Genesee Scientific 20-304), goat anti-rabbit–HRP (Genesee Scientific 20-303), and SuperSignal West Dura Extended Duration Substrate (Thermo Fisher Scientific 34075) were used to visualize blots on a Bio-Rad GelDoc imager.

### Stop codon reporter

mCherry was removed via restriction digest from the Artichoke plasmid (Addgene 73320). BFP was amplified using standard Phusion PCR protocol. Primers were designed to introduce BsmBI (New England Biolabs) restriction sites at C- and N-termini, and the reverse primer sequence was altered to introduce the different stop codons. PCR products were purified using the QIAquick PCR Purification Kit (Qiagen), digested with BsmBI (New England Biolabs), and ligated into Artichoke (Addgene 73320) reporter vector using the T4 DNA ligase (New England Biolabs).

Virus was made as described above and transduced into K562 cells. Cells were seeded into 96-well plates at 2 × 10^4^ cells per well. Cells were treated with G418 or CC-885 at the indicated doses. Cells were assessed by flow cytometry at 24, 48, and 72 hours with live cells defined by forward and side scatter and the MFI of BFP and GFP measured. Flow cytometry was performed on a FACSCanto II (BD Biosciences).

### RNA sequencing

One million cells were collected at the indicated time points following treatment with DMSO, CC-885, or CC-90009. RNA was extracted using an RNeasy kit (Qiagen 74106). The quality and concentration of extracted RNA were determined using a NanoDrop Spectrophotometer (Thermo Fisher Scientific) and RNA ScreenTape (Agilent). RNA integrity number equivalent (RINe) values greater than 8.0 were considered acceptable. One microgram of total RNA was sent for sequencing by Novogene using its paired-end 150 service. RNA-Seq data were aligned with STAR (RRID:SCR_004463) (38) and the differential expression determined by Cufflinks (RRID:SCR_014597) (39). RNA-Seq data were deposited in the NCBI’s Gene Expression Omnibus repository (GEO GSE206223).

### Global quantitative proteomics on native 293T cells or 293T cells overexpressing GSPT1 or GSPT1^G575N^

Quantitative proteomic profiling by liquid chromatography–tandem mass spectrometry (LC-MS/MS) was performed on HEK293T cells, as well as HEK293T cells expressing GSPT1 or GSPT1^G575N^. Cells were treated for 18 hours with 1 μM CC-885 or DMSO with 2 biological duplicates for all conditions. Harvested cells were treated as previously described (40). Tandem mass tag–labeled (TMT-labeled) peptides were fractionated by offline basic reverse-phase chromatography into 24 fractions as previously described (41). MS/MS data were processed using Spectrum Mill version BI.07.04.210 (Agilent Technologies, Broad Institute of MIT and Harvard), and protein quantitative values were further analyzed using R. Further details on proteomic sample processing, LC-MS/MS conditions, and data analysis are provided in Supplemental Methods.

### Analysis of *TP53*-mutant isogenic cell lines

Isogenic MOLM13 cell lines have been previously described (25). Twenty thousand cells were seeded per well in a 96-well flat-bottom plate. The following compounds were used: daunorubicin (SelleckChem S3035), cytarabine (SelleckChem S1648), and CC-885 (MedChemExpress HY-101488). Drugs were added to wells in limiting dilutions with a Tecan D300e Digital Dispenser. After 72 hours of drug exposure, cell viability was assessed by CellTiter-Glo luminescent assay (Promega G7572) using a FilterMax F5 (Molecular Devices) plate reader. Cell viabilities were calculated relative to DMSO controls.

### Bison screen

The Bison CRISPR screen was performed as previously described and cells harvested and processed 4 hours after treatment with 1 μM CC-885 or DMSO (27, 28). Further details are provided in Supplemental Methods.

### Genome-wide CRISPR screen

MM.1S cells expressing Cas9 were spinfected with the Human Avana LentiGuide-Puro library (Broad Genetic Perturbations Platform) in the presence of 2 μg/mL Polybrene. Twenty-four hours after infection, cells were selected with 1 μg puromycin/mL for 2 days. Cells were expanded for 12 days, and then on day 0, 40 million cells were plated for DMSO treatment, and cells were treated with 0.25 nM CC-885. Cells were passaged and redosed every 3–4 days with DMSO or 0.25 nM CC-885. On day 16, more than 60 million cells from the DMSO arm and the CC-885 arm were pelleted. The sgRNA library was PCR-amplified from genomic DNA isolated from the cell pellets, and the resulting amplicons were sequenced on an Illumina NovaSeq SP. Data were analyzed as previously described (27, 28).

### CRISPR screen validation

sgRNAs targeting genes of interest were cloned into sgRNA.SFFV.tBFP, sgRNA.SFFV.tRFP, or sgRNA.SFFV.RFP657 flourescent vectors using BsmBI digestion as previously described (42). In brief, vectors were linearized with BsmBI (New England Biolabs) and gel-purified (Qiagen Spin MiniPrep). Annealed oligonucleotides were phosphorylated with T4 polynucleotide kinase (New England Biolabs) and ligated into linearized vector backbone. Constructs were transformed into XL10-Gold ultracompetent *E*. *coli* (Stratagene or Agilent Technologies), and plasmids were purified using the MiniPrep Kit (Qiagen) and validated by Sanger sequencing. Lentivirus was produced as described above. Cells were then spinfected with lentivirus containing sgRNAs. The percentage of fluor-positive cells was monitored over time by flow cytometry after the addition of DMSO or drug.

### Generation of Ba/F3 humanized Crbn constructs

HA-Crbn^V380E^ and HA-Crbn^I391V^ retrovirus plasmids were previously generated (43). Combinations of HA-Crbn with S105F, E152D, V380E, and I391V were generated using site-directed mutagenesis, sequence-verified gBlocks (Integrated DNA Technologies), and overlapping primers. Retrovirus was made using Ecopak packaging plasmid and transfected into HEK293T cells using Mirus Bio TransIT-LT1 Transfection Reagent (Thermo Fisher Scientific). Viral supernatant was collected 48 hours after transfection, cleared by centrifugation at 500*g* for 5 minutes, and then filtered through a Millex-HV Syringe Filter Unit, 0.45 μm (MilliporeSigma). Ba/F3 cells were spinfected at 800*g* for 90 minutes in tissue culture plates coated with RetroNectin (Takara). After 24 hours, cells were dissociated and cultured in RPMI supplemented with 100 U/mL penicillin and 100 mg/mL streptomycin with 10% FBS, and mouse IL-3 (10 ng/mL; PeproTech), in a 37°C incubator at 5% carbon dioxide. After 48 hours, cells were selected with puromycin (4 μg/mL) for 96 hours.

For immunoblots, Ba/F3 cells containing constructs were treated with CC-885 or lenalidomide at the indicated doses, harvested after 6 hours, and processed as above. Antibodies used for immunoblotting were Gspt1 (Abcam), Csnk1a1 (Cell Signaling Technology), HA (Miltenyi Biotec), and β-actin (Abcam). Goat anti-mouse–HRP (Genesee Scientific 20-304), goat anti-rabbit–HRP (Genesee Scientific 20-303), and SuperSignal West Dura Extended Duration Substrate (Thermo Fisher Scientific 34075) were used to visualize the blot on a Bio-Rad GelDoc imager.

For cell survival studies, 2 × 10^4^ Ba/F3 cells were treated with drug at the indicated doses and assessed by CellTiter-Glo as above.

### Generation of *Crbn^V380E/I391V^* mice

*Crbn^V380E/I391V^* mice were generated via homologous recombination by Ingenious Targeting Laboratory on a C57BL/6 background. The FLP recombinase target (FRT) sites and neomycin cassette were removed by crossing with C57BL/6 FLP mice. Our mouse has been accepted and deposited in the JAX Repository at The Jackson Laboratory (stock 035831 Crbn<V380E/I391V>).

Isolation of peripheral blood and blood counts

Peripheral blood was obtained via retro-orbital bleeds. Blood counts were obtained from a Hemavet (Drew Scientific) or Element HT5 Analyzer (Heska Corp.) using mouse settings. The percentage of B220^+^, CD3^+^, CD11b^+^, and Gr1^+^ cells in erythrocyte-lysed peripheral blood was determined by flow cytometry on a FACSCanto II (BD Biosciences).

### Isolation of bone marrow and c-Kit+ cells

A single-cell suspension of bone marrow was obtained by crushing of long bones and pelvis with a mortar and pestle. Resuspension in RBC Lysis Solution (Qiagen) was used to lyse erythrocytes, except for Ter119/CD71 staining. c-Kit^+^ cells were isolated with CD117 microbeads (Miltenyi Biotec) and an AutoMACS Pro (Miltenyi Biotec) and grown in Serum-Free Expansion Media (StemCell Technologies) supplemented with 50 ng/mL mouse thrombopoietin (TPO; PeproTech) and 50 ng/mL mouse stem cell factor (SCF; PeproTech).

### Generation of Mll-Af9 leukemia

Retrovirus was generated using the pMIG *Mll-Af9* GFP vector using Ecopak packaging plasmid and TransIT-LT1 as described above. Isolated c-Kit^+^ cells from *Crbn^WT^*, *Crbn^I391V^*, and *Crbn^V380E/I391V^* mice were prestimulated with mouse TPO (50 ng/mL) and mouse SCF (50 ng/mL) in StemSpan Serum Free Expansion Media (SFEM) (STEMCELL Technologies). After 48 hours, c-Kit^+^ cells were spinfected with virus onto plates coated with RetroNectin (Takara) at 800*g* for 90 minutes. After 24 hours, cells were dissociated and washed in PBS, and 50,000 cells were transplanted by retro-orbital injection into sublethally irradiated C57BL/6 mice (475 cGy 4 hours before transplant). For *Tp53-*null *Mll-Af9* leukemia, *Crbn^V380E/I391V^* mice were crossed with *Trp53* (*Tp53*-null) mice and homozygous mice were genotyped and selected before generation of *Mll-Af9* leukemia as above.

At the onset of leukemia, bone marrow and spleen from individual mice were isolated and viably frozen (90% FBS plus 10% DMSO) for future transplant experiments. Cells were also continued in culture with RPMI supplemented with 100 U/mL penicillin and 100 mg/mL streptomycin with 10% FBS, and mouse IL-3 (10 ng/mL), in a 37°C incubator at 5% carbon dioxide.

### Assessment of *Mll-Af9* leukemias generated from *Crbn^WT^*, *Crbn^I391V^*, and *Crbn^V380E/I391V^* mice

#### In vitro survival analysis.

In vitro survival analysis was performed 72 hours after treatment with CC-885, CC-90009, or omacetaxine at the indicated doses. Viability was read using CellTiter-Glo as above or by counting of viable cells per well using flow cytometry.

#### Immunoblots.

Cells were treated for 6 hours with CC-885, CC-90009, or lenalidomide at 1 μM and then processed and immunoblotted as above.

### Global quantitative proteomics on mouse cells expressing Crbn variants

c-Kit^+^ magnetic bead–selected (Miltenyi Biotec) primary bone marrow cells from C*rbn^WT^*, *Crbn^I391V^*, and *Crbn^V380E/I391V^* mice were treated with DMSO or 0.1 μM CC-885, 1 μM CC-90009, or 1 μM pomalidomide in biological duplicate for 6 hours. Mll-Af9 cells containing either *Crbn^WT^*, *Crbn^I391V^*, or *Crbn^V380E/I391V^* were treated with DMSO or 0.1 μM CC-885, 1 μM CC-90009, or 1 μM pomalidomide in biological replicates for 6 hours. All cells were harvested by centrifugation and washed with PBS. Full details of mouse proteomics are given in Supplemental Methods (44–46).

#### Reverse transcriptase PCR.

One million cells were collected after 24 hours of treatment with DMSO, CC-885, or CC-90009. RNA was extracted using an RNeasy kit (Qiagen 74106). The concentration of extracted RNA was determined using a NanoDrop Spectrophotometer (Thermo Fisher Scientific). One hundred nanograms of total RNA was used for cDNA synthesis using a QuantiTect Reverse Transcription kit (Qiagen). Transcript abundance was determined using TaqMan probes targeting Atf3 (Mm00626978_m1, Life Technologies) and Gapdh (Mm99999915_g1, Life Technologies) with TaqMan Gene Expression Master Mix (Life Technologies). Reactions were run and analyzed on the QStudio 6 FLX Real-Time PCR System (Thermo Fisher Scientific).

#### In vivo survival analysis.

Secondary *Mll-Af9* *Crbn^V380E/I391V^* leukemia cells were treated at the 0.01 μM dose of CC-885 (approximate IC_90_) or DMSO in RPMI supplemented with 100 U/mL penicillin and 100 mg/mL streptomycin with 10% FBS, and mouse IL-3 (10 ng/mL), in a 37°C incubator at 5% carbon dioxide. After 5 hours, cells were washed 3 times in PBS. Cells were transplanted by retro-orbital injection into sublethally irradiated *Crbn^WT^* C57BL/6 recipient mice (475 cGy 4 hours before transplant). Mice were randomized using the rand() function of Excel (Microsoft) into 2 groups (*n* = 15 per group DMSO or CC-885 treated) and received 10,000 live cells. Disease burden was assessed by sampling of peripheral blood, erythrocyte lysis as above, and assessment of percentage GFP^+^ cells on a BD Biosciences FACSCanto II. Mice were followed for up to 65 days and were euthanized when they became moribund.

For in vivo leukemia treatment, 10,000 secondary *Mll-Af9* *Crbn^V380E/I391V^* leukemia cells were transplanted by retro-orbital injection into sublethally irradiated *Crbn^WT^* C57BL/6 recipient mice (475 cGy 4 hours before transplant). Mice were randomized into treatment or control group using the rand() function of Excel (Microsoft). Mice were bled weekly and the percentage of GFP^+^ cells determined by flow cytometry. After 2 weeks, when GFP^+^ cells first became apparent, mice were treated with CC-90009 (MedKoo) at a dose of 20 mg/kg once daily or DMSO by oral gavage. Mice were bled weekly and the percentage of GFP^+^ cells determined by flow cytometry. At the conclusion of the experiment, mice were euthanized, and the percentage of GFP^+^ cells in the spleen and bone marrow was determined by flow cytometry.

#### In vivo competition experiments.

A 50:50 mix of bone marrow from *Crbn^V380E/I391V^* CD45.2 and C57BL/6.SJL CD45.1 mice was prepared. Two million cells were transplanted by retro-orbital injection into lethally irradiated *Crbn^WT^* C57BL/6.SJL CD45.1 recipient mice (950 cGy in 2 split doses 4 hours before transplant). Following hematopoietic reconstitution at 6–8 weeks after transplant, chimerism was confirmed using flow cytometry of peripheral blood with antibodies recognizing CD45.1 and CD45.2. Mice were randomized as before and treated with CC-90009 (MedKoo) or DMSO at the indicated doses and times. Peripheral blood chimerism was monitored weekly. At the completion of the experiment, mice were euthanized, and the chimerism in peripheral blood and bone marrow compartments was determined using flow cytometry on a BD Biosciences FACSCanto II.

#### Stem cell and spleen analysis.

Cellular suspensions of bone marrow or spleen were made by crushing and filtering through a 40 μm filter. Resuspension in RBC Lysis Solution (Qiagen) was used to lyse erythrocytes, except for Ter119/CD71 staining. Cells were then resuspended in PBS supplemented with 5% FBS and stained with indicated antibodies or compensation controls. Analysis was performed on a BD Biosciences FACSCanto II.

#### Secondary transplants.

Whole bone marrow containing *Crbn^V380E/I391V^* CD45.2 and C57BL/6.SJL CD45.1 cells was prepared from the 50:50 chimeric mice treated with either vehicle control or CC-90009 (mice from Figure 6) and transplanted into lethally irradiated C57BL/6.SJL CD45.1 recipients. Transplants were performed as described above. Chimerism and cell compartments were assessed, as described above, at 5, 9, 13, and 15 weeks after transplant.

### Statistics

Protein summaries were generated using “subgroup top grouping,” and ratio data were calculated as each TMT reporter ion intensity over the mean of the 2 control replicates (native 293T cells treated with DMSO). Subsequent statistical analysis was done using R/Protigy. The stop codon reporter analysis was performed using Kruskal-Wallis or Wilcoxon’s tests as indicated. For animal experiments, differences between groups were assessed using 1- or 2-tailed unpaired *t* test as indicated.

### Study approval

Animal work was carried out according to protocols ethically approved by the IACUCs at Brigham and Women’s Hospital and the Dana-Farber Cancer Institute.

## Author contributions

RSS and BLE initiated the project. LY, AC, SPP, and CWC designed and performed the CRISPR dropout screen. RSS, ASS, MS, and JK designed and performed the other CRISPR screens. RSS, EJB, RB, NDU, and SAC performed human proteomics experiments. ASS, KAD, NM, and ESF performed mouse proteomics experiments. SB generated isogenic cell lines. RSS, ECF, and BLE designed the mouse model. RSS and ASS performed functional validation studies and analysis of the mouse model with help from JAG, MEM, DNA, CZ, PGM, AEL, BS, RKD, VAQB, EKH, and VK. RSS, ASS, MS, and BLE wrote the manuscript with input from all authors. RSS and ASS contributed equally to the experiments and analysis. Authorship order was decided based on RSS initiating the project.

## Supplementary Material

Supplemental data

Supplemental table 1

Supplemental table 2

Supplemental table 3

Supplemental table 4

Supplemental table 5

Supplemental table 6

Supplemental table 7

## Version 1 06/28/2022

In-Press Preview

## Version 2 08/15/2022

Electronic publication
