# Supplementary material for: Degradation of GSPT1 causes TP53-independent cell death in leukemia while sparing normal hematopoietic stem cells
Source: J Clin Invest. 2022 Aug 15;132(16):e153514. doi: 10.1172/JCI153514 (PMC9374383; doi:10.1172/JCI153514)
Supplement: Supplemental data [file jci-132-153514-s083.pdf]

Supplementary Figure 1

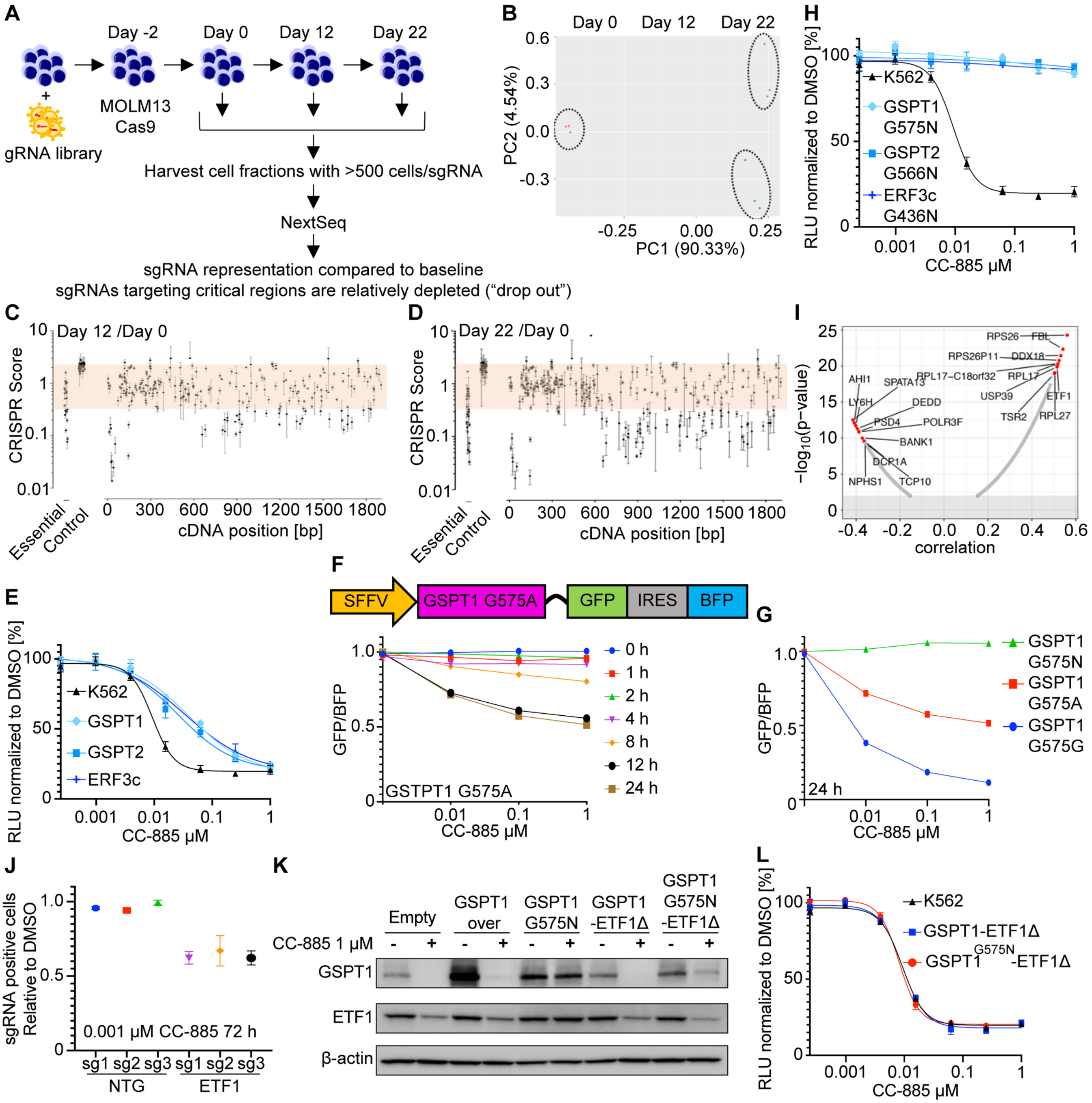

Supplementary Figure 1. Design and results of GSPT1 tiled CRISPR-Cas9 depletion screen. A. Overall design of tiled GSPT1 screen. **B.** Analysis of sgRNA representation clustering of triplicate replicates from day 0, day 12, and day 22. **C, D.** Representation of location of individual sgRNAs (x-axis numbers refer to nucleotide position in coding sequence), and relative representation at day 12 versus day 0 (C) and day 22 versus day 0 (D). Individual points represent the median, and error bars the limits, of the results from 3 individual replicates. **F.** Dose and time dependent effects of CC-885 treatment on GSPT1(G575A). **G.** Comparison of CC-885 induced degradation of GSPT1, GSPT1(G575A), and GSPT1(G575N). **H.** Cell viability of K562 cells expressing wild-type or drug resistant constructs. Cell viability assessed using CellTiter-Glo luminescent assay 72 hours after treatment with CC-885 (replicates, n = 3; symbols represent mean ± SEM). **I.** Analysis of cell lines from Broad Institute DepMap (Avana CRISPR libraries) showing top co-dependencies (right side) with GSPT1 loss. **J.** Effects of sgRNAs directed against ETF1 and treatment with CC-885 in K562 cells. Ratios represent the number of guide positive (RFP657) cells relative to DMSO treated cells. Data is representative of 3 biological replicates. Points represent the mean and SEM for 3 technical replicates. **K.** Western blot analysis of total GSPT1 and ETF1 in MOLM13 cells with overexpression construct as indicated **L.** Cell viability of K562 cells expressing GSPT1-ETF1BΔ (drug sensitive and drug resistant – G575N). For all CellTiter-Glo luminescent assays, cell viability was assessed assay 72 hours after treatment with CC-885 (figures represent combined data from 3 biological replicates performed in technical triplicates; symbols represent mean ± SEM).

Supplementary Figure 2

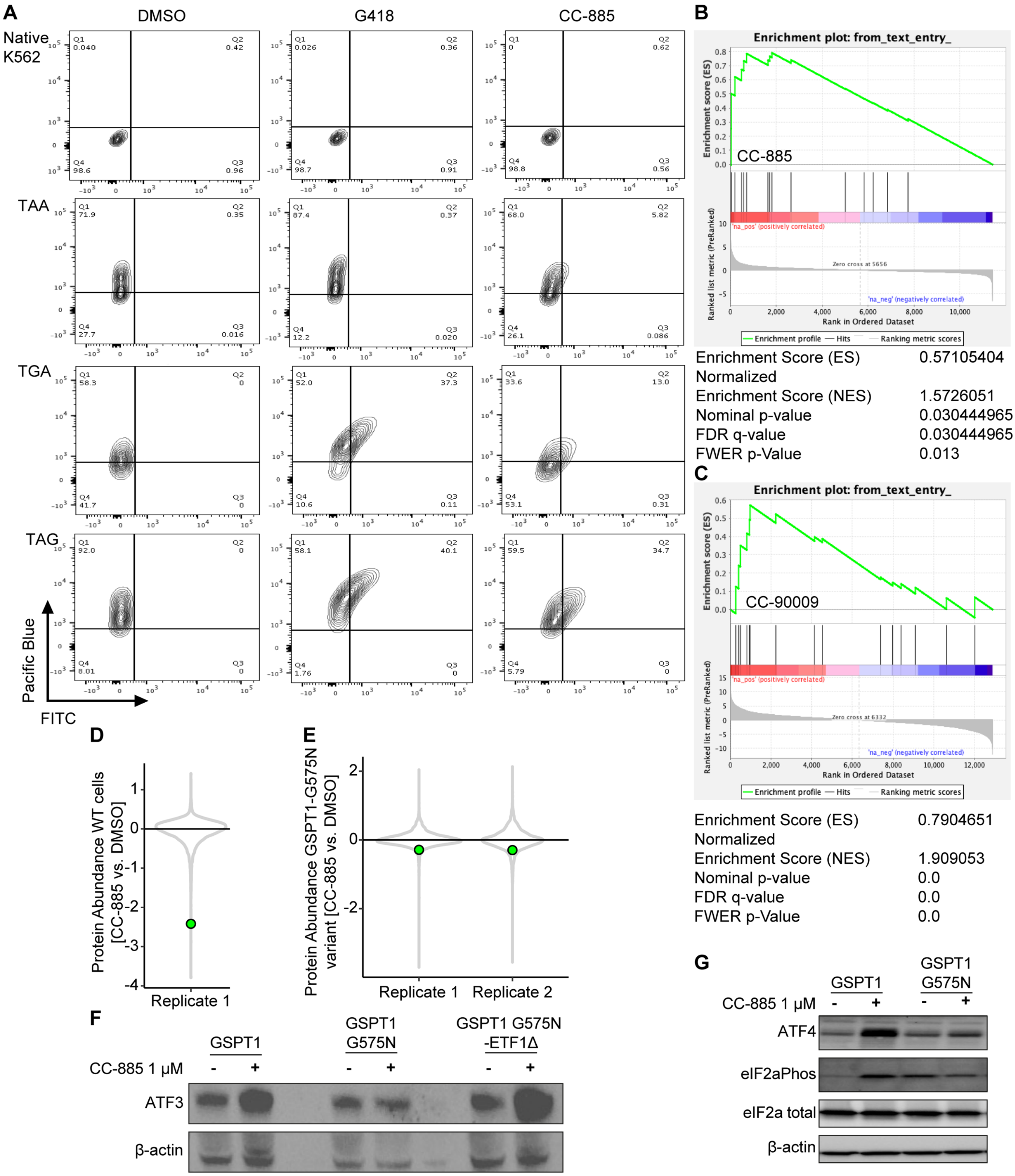

Supplementary Figure 2. A. Representative plots of stop codon BFP stop codon reporter in K562 cells 48 hours after treatment with G418 (2mg/ml) or CC-885 (1mM). TAA, TGA, TAG, indicates the stop codon at the end of BFP. **B.** GSEA for genes in ATF4 pathway performed on RNAseq data from MOLM13 cells treated for 6 hours with 1mM CC-885. **C.** GSEA for genes in ATF4 pathway performed on RNAseq data from MOLM13 cells treated for 6 hours with 1mM CC-90009. Genes enriched: *ASNS, ATF3, ATF4, ATF6, DDIT3, DIS3, DNAJB9, ERN1, EXTL1, HERPUD1, HSPA5, PDIA5, PPP2R5B, TLN1, TSPYL2, XBP1.* **D.** Relative abundance of GSPT1 (shown in green on violin plots) in the proteomics data for 293T cells expressing WT GSPT1, measured in singlicate, and (**E)** 293T cells expressing GSPT1^{G575N}, measured in duplicate. Protein abundance is represented by TMT-reporter ion intensity ratios, following 18 hours treatment with CC-885. **F.** Western blot for ATF3 in K562 cells expressing indicated construct following treatment with CC-885 or control. **G.** Western blot of whole lysates prepared from HEK293T cells overexpressing GSPT1 or GSPT1^{G575N} 8 hours following treatment with DMSO or 1mM CC-885.

Supplementary Figure 3

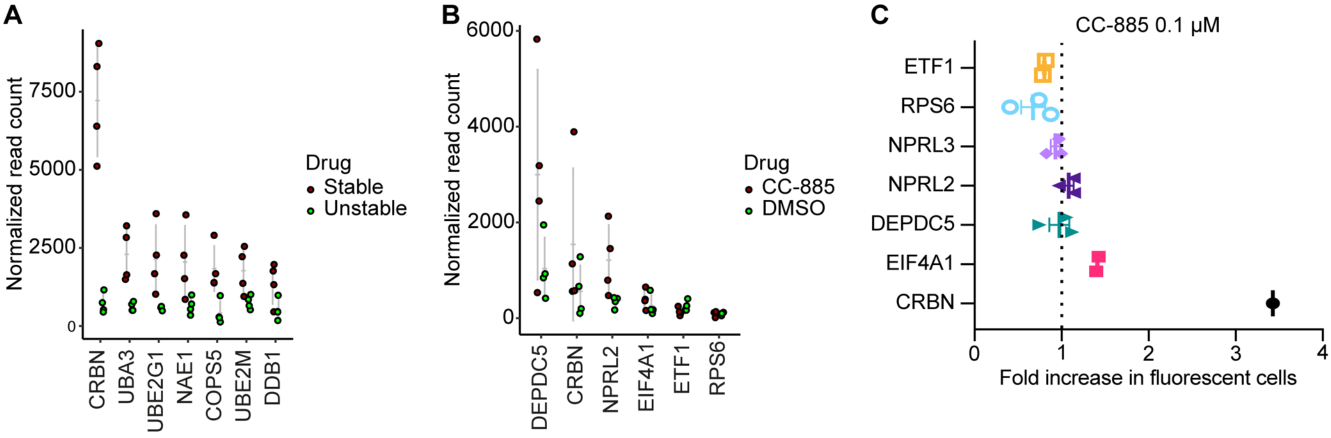

Supplementary Figure 3. A. Bison CRISPR–Cas9 knockout screen for GSPT1 stability treated with CC-885 relative to DMSO in HEK293T cells. Normalized read counts in each sorted gate for 4 sgRNAs targeting genes highlighted in Figure 3A. Symbols indicate the mean normalized read numbers for each sgRNA (n = 3). **B.** Genome-wide CRISPR–Cas9 knockout screen for CC-885 resistance in MM1S cells. Normalized read counts in each sorted gate for 4 sgRNAs targeting genes highlighted in Figure 3C. Symbols indicate the mean normalized read numbers for each sgRNA (n = 3). **C.** Selected sgRNA from arrayed validation screen in MM1S cells showing fold change in representation following 4 days treatment with 0.1μM CC-885.

Supplementary Figure 4

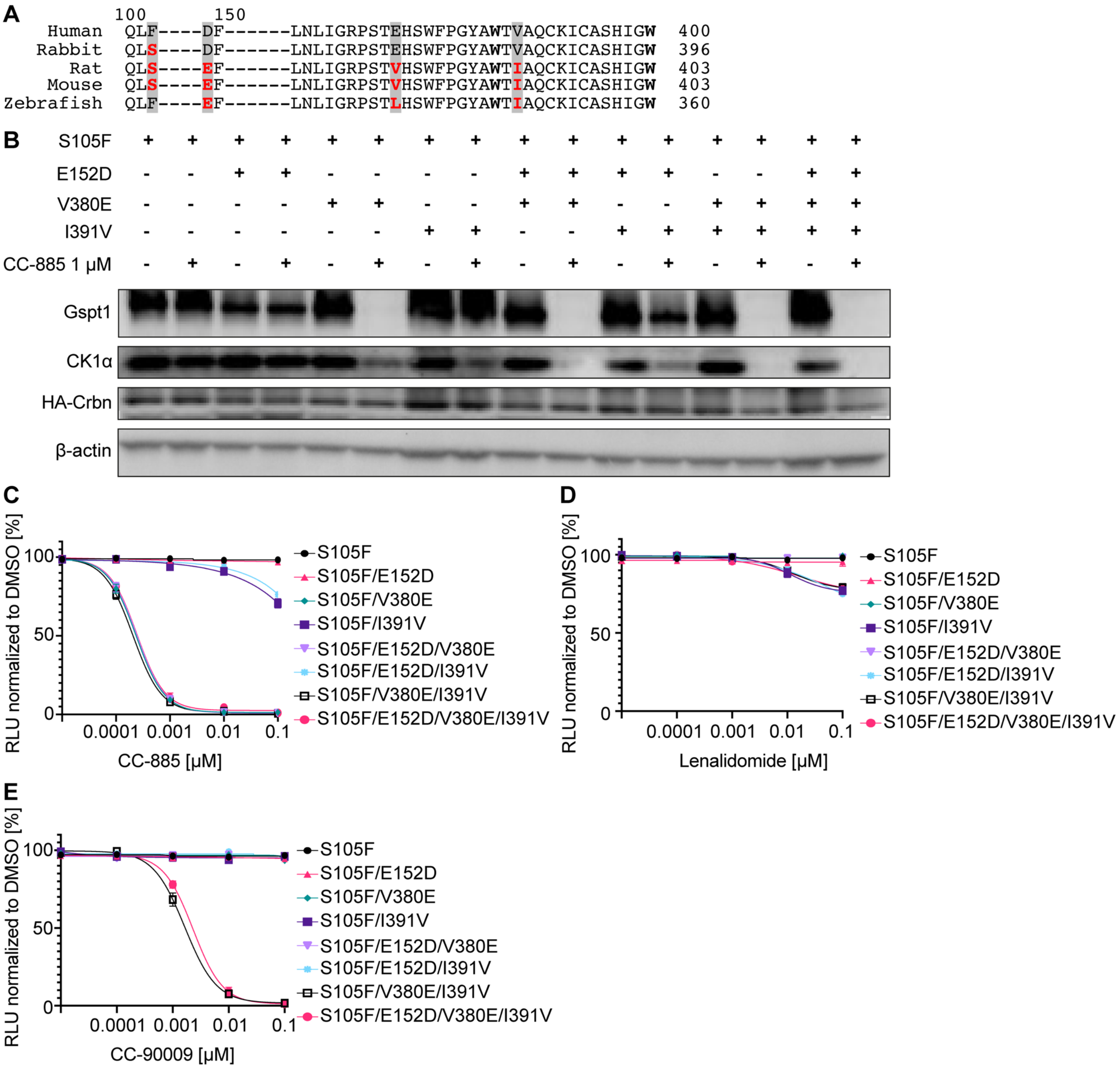

Supplementary Figure 4. A. Comparison of amino acid sequence of drug binding surface of CRBN across species. Amino acid positions 100 and 150 are numbered for the human sequence. Red letters indicate the non-conserved amino acids between human and mouse. **B.** Western blot of lysates from Ba/F3 cells overexpressing Crbn constructs with combinations of humanization at S105F, E152D, V380E, and I391V as indicated. Protein lysates were harvested following 6 hours treatment with 1μM CC-885. **C-E.** Cell viability of Ba/F3 overexpressing Crbn constructs with humanization mutations following treatment with CC-885 (C), Lenalidomide (D), or CC-90009 (E). Cell viability assessed using CellTiter-Glo luminescent assay 72 hours after treatment (figures represent combined data from 3 biological replicates performed in technical triplicates; symbols represent mean ± SEM).

Supplementary Figure 5

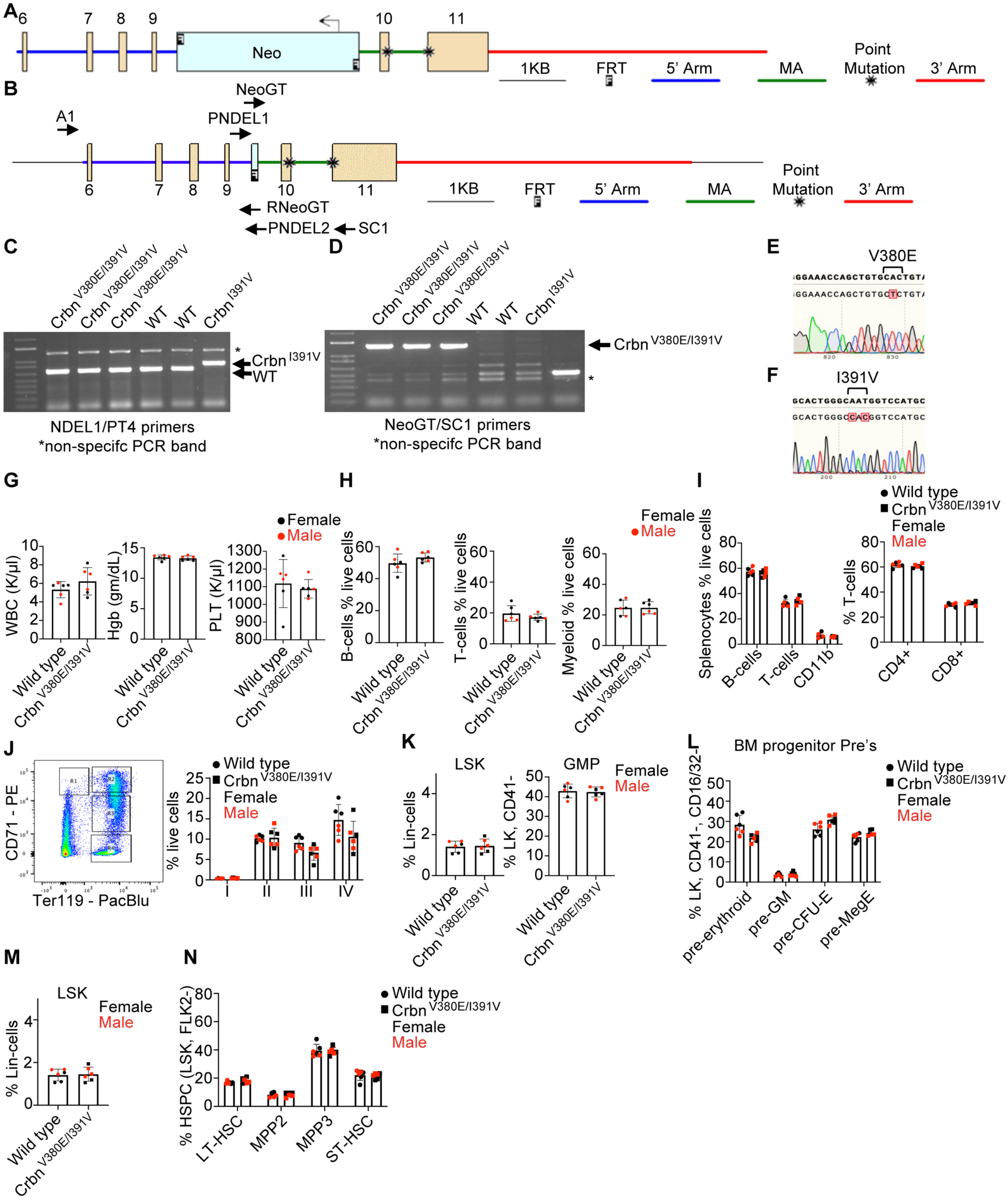

Supplementary Figure 5 continued

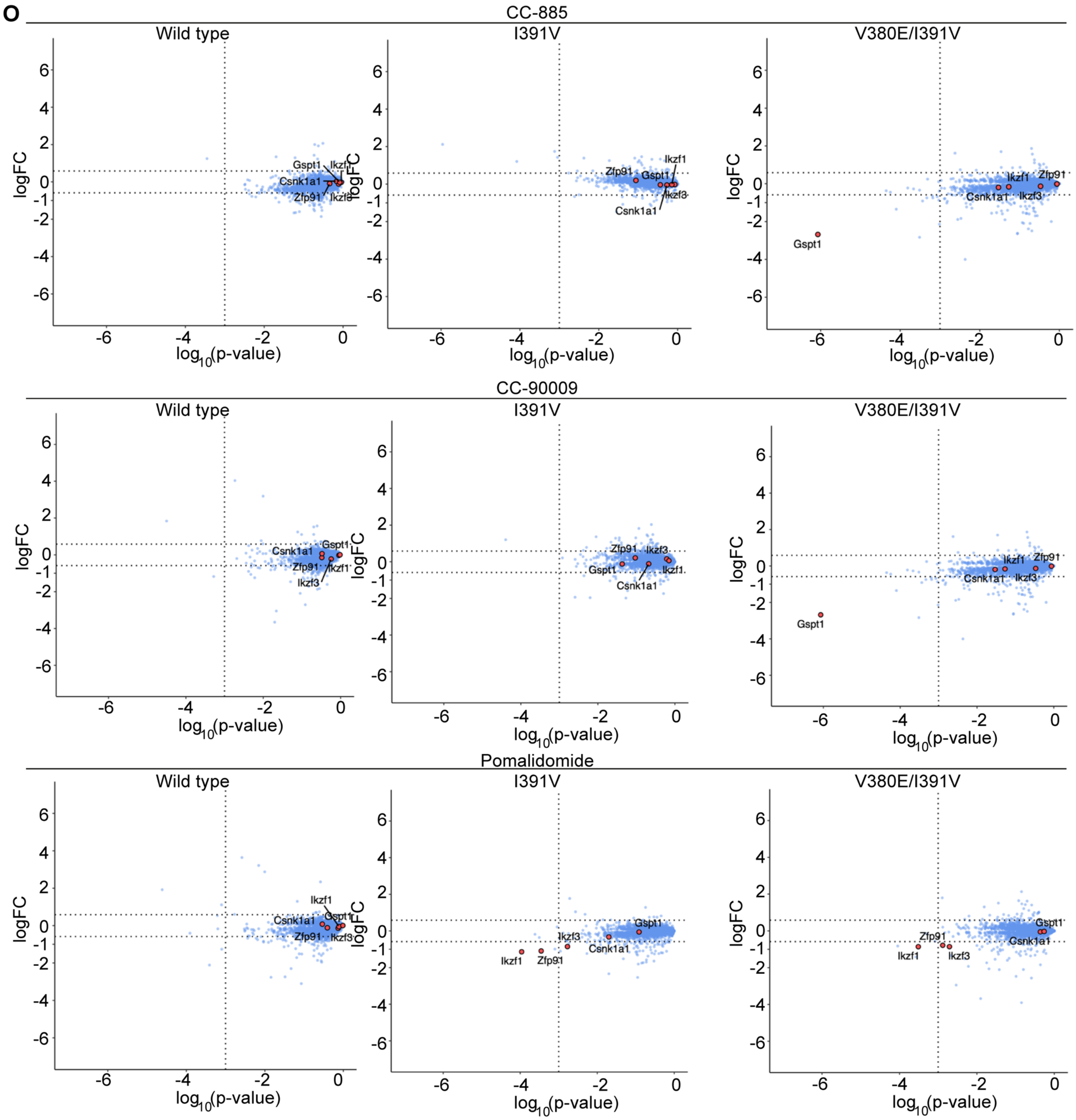

Supplementary Figure 5 continued

P

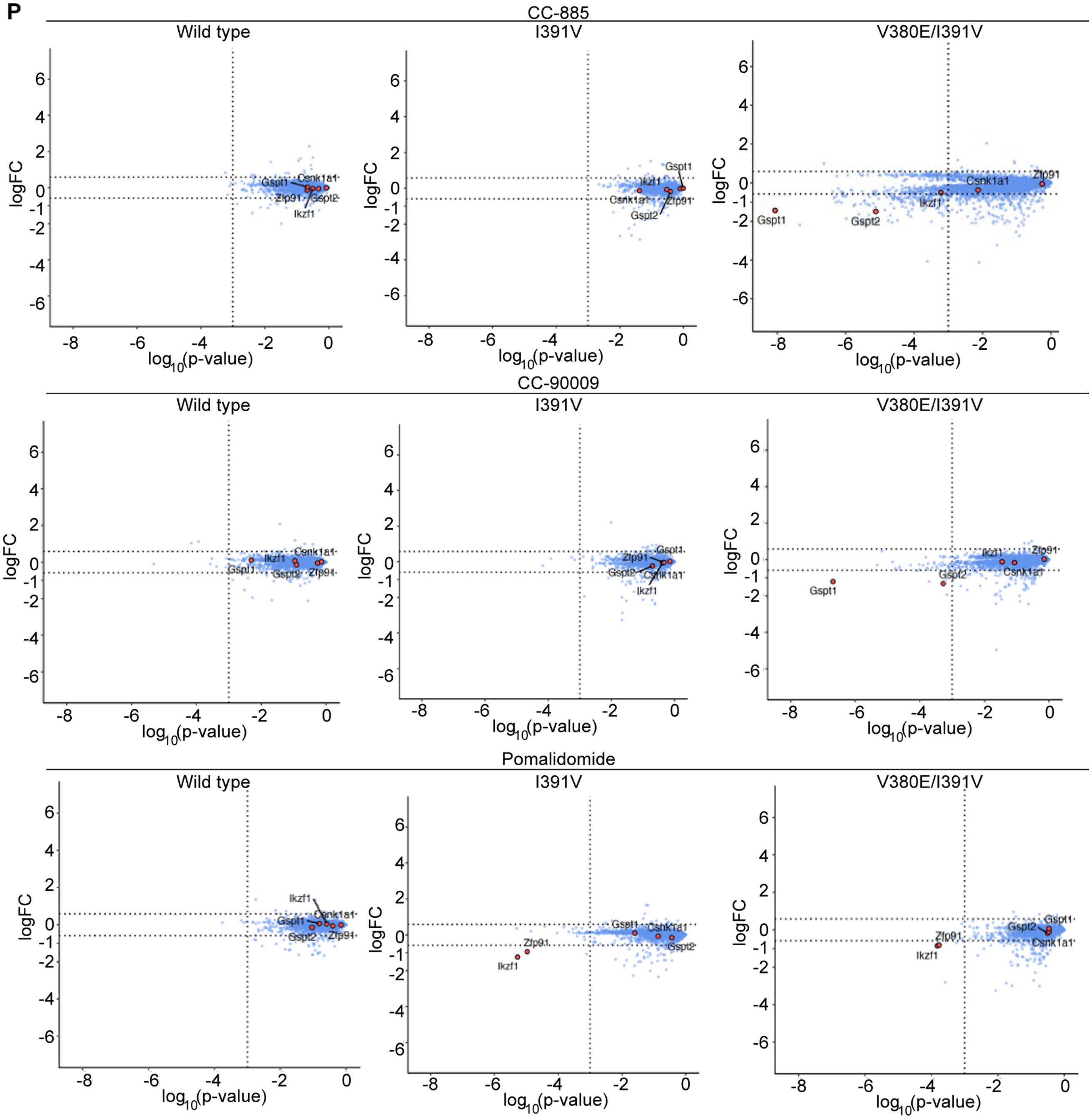

Supplementary Figure 5 continued

Supplementary Figure 5. Design and confirmation of double humanized Crbn$^{V380E/I391V}$ mouse. A,B. Schematic of *Crbn* locus and target construct before and after removal of Neomycin cassette. Location of targeting primers is indicated **C.** PCR using genotyping primers for *Crbn^{I391V}* mouse from Fink et al. Blood (2019)[19]. **D.** PCR using genotyping primers for *Crbn$^{V380E/I391V}$*. **E, F.** Sanger sequencing traces from *Crbn$^{V380E/I391V}$* mouse (NeoGT/SC1 amplicon) using SC1 primer. Wild type (WT) sequence is shown on top track with the targeted codons highlighted. **G.** Peripheral blood counts of total white cells (WBC), Hemoglobin (Hgb), and platelets (Plt) in wild-type or *Crbn$^{V380E/I391V}$* mice as indicated. Data points for female mice are shown in black, data points for male mice are shown in red. **H.** Flow cytometry assessment of B-cells (B220^{+}), T-cell (CD3^{+}), and myeloid cell (CD11b^{+}) composition of white blood cell compartment. **I.** Quantification of B-cells (CD220^{+}), T-cells (CD3^{+}), and myeloid (CD11b^{+}) cells from splenocytes according to genotype and sex (left) and assessment of CD4^{+} and CD8^{+} positive cells in the T-cell population. **J.** Assessment of erythroid differentiation as assessed by expression of CD71 and Ter119 on bone marrow cells. **K.** Assessment of LSK and GMP bone marrow cells. GMP are Lineage^{-}, c-Kit^{+}, Sca1^{-}, CD16/32^{hi}. LSK are Lineage^{lo}, c-Kit^{+}, Sca1^{+}. Lineage markers used CD3, B220, CD11b, Gr1, and Ter119. **L.** Quantification of Lineage^{lo}, c-Kit^{+}, CD41^{-} bone marrow cells according to pre-erythroid (CD16/32^{-}, CD105^{+}, CD150^{-}), pre-CFU-E (Colony Forming Unit-Erythroid; CD16/32^{-}, CD105^{+}, CD150^{+}), pre-GM (pre-granulocyte-macrophage progenitor; CD16/32^{-}, CD105^{-}, CD150^{-}), and pre-MegE (pre-megakaryocyte-erythroid progenitor; CD16/32^{-}, CD105^{-}, CD150^{+}). **M.** Quantification of bone marrow LSK cells that are MPP4 (multipotent progenitor 4; CD135^{+}) or HSPC hematopoietic stem progenitor cells. **N.** Quantification of bone marrow HSPCs (LSK, CD135^{-}) that are LT-HSC (long-term hematopoietic stem cells; CD48^{-}, CD150^{+}), MPP2 (multipotent progenitor 2; CD48^{+}, CD150^{+}), MPP3 (multipotent progenitor 3; CD48^{+}, CD150^{-}), or ST-HSC (short-term hematopoietic stem cells; CD48^{-}, CD150^{-}). Differences between groups were assessed using 2-tailed unpaired t-Test. All differences were statistically non-significant at a cut off of $p=0.05$. **O.** Example proteomics for wild-type, I391V, and V380E/I391V Crbn mice following treatment with CC-885, CC-90009, or pomalidomide. **P.** Example proteomics for *Mll-Af9* wild-type, I391V, and V380E/I391V Crbn mice following treatment with CC-885, CC-90009, or pomalidomide.

Supplementary Figure 6

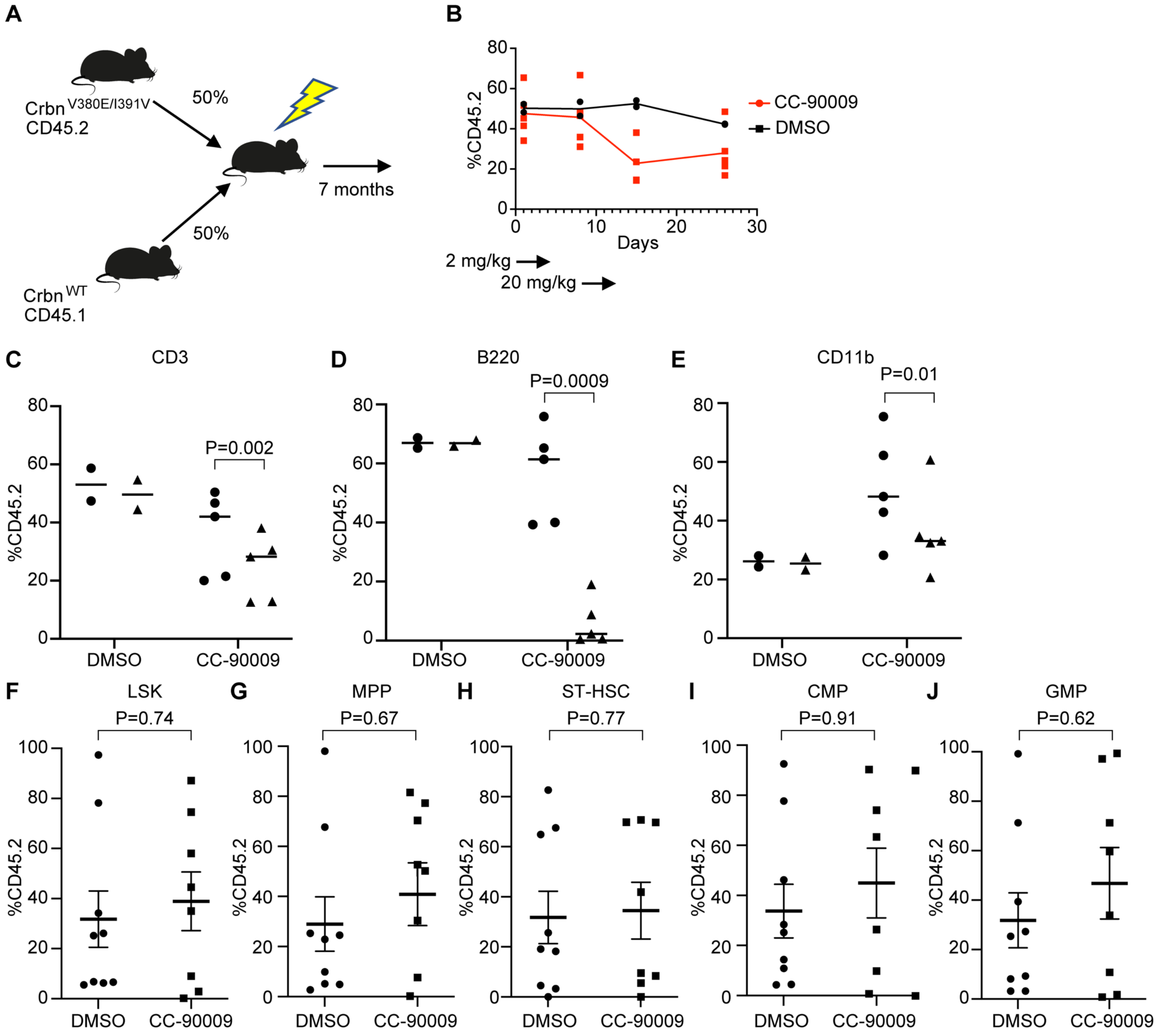

Supplementary Figure 6. *In vivo* treatment with CC-90009 selectively targets *Crbn*$^{V380E/I391V}$ cells. A. Design of mixed chimera mice transplanting c-Kit selected cells, 50% *Crbn*$^{V380E/I391V}$ (CD45.2) and 50% *Crbn*^{WT} (wild type, CD45.1) following irradiation of recipient animal. **B.** % chimerism in peripheral blood of CD45.2 *Crbn*$^{V380E/I391V}$ cells following 5 days treatment with CC-90009 at 2mg/kg or 20mg/kg as indicated. **C,D,E.** % chimerism of CD45.2 cells in CD3, B220, and CD11b positive cells before (circles) and following (triangles) treatment with CC-90009 at 20mg/kg. **F-J.** %chimerism of CD45.2 cells assessed at 15 weeks following secondary transplantion in stem and progenitor cells as indicated. Lineage markers used to define these are as described for Figure 6 and 6S, and in materials and methods.

SUPPLEMENTAL METHODS

Tiled CRISPR library

Oligo pools (CustomArray) were diluted to a final concentration of 10ng/ µl and PCR amplified using specific forward and reverse primers and the NEBNext Ultra II Q5 kit (NEB). PCR product was gel purified before BsmBI digestion, and ligation into linearized sgRNA vector (ipUSEPR). Ligated plasmids were used to transform Endura Electrocompetent cells (Lucigen Cat#60242-2) and the BioRad GenePulser. Cells were plated in a BioAssay Dish (Corning #43111) containing LB agar with ampicillin and serial dilutions on 10cm petri dishes and incubated for 16 hours at 37 ºC. Serial dilutions were used to estimate the number of colonies in the BioAssay dish (aiming for at least 100X coverage of the total library). Colony lawn harvested using a cell scraper, incubated in LB medium with ampicillin, and shaken at 250rpm for 60mins at 37 ºC. Cells were pelleted and then plasmids harvested using one Promega Maxiprep kit per 0.45g wet cells. Guide representation of the library was assessed (QC) using NextSeq.

The plasmid library was used to make lentivirus using packaging plasmids with VSV-G (Addgene #12259) and psPAX2 (Addgene #12260) and using TRANS-LTI (Mirrus) into HEK293T cells. The viral supernatant was collected 48 h after transfection, cleared by centrifugation at 500 g for 5 min, and then filtered through a Millex-HV Syringe Filter Unit, 0.45 µm (EMD Millipore). Serial dilutions of virus were used to transduce MOLM13 cells to determine virus concentration giving a transduction efficiency (as assessed by RFP positive cells) of 15-20%, 48 hours following spinfection (800g for 90 mins). MOLM13 cells expressing CAS9 (Broad Institute cell line repository) were re-selected using blasticidin (10 µg/ml for 10 days) and then transduced in triplicate (Day -2).

Bison screen

The Bison CRISPR library targets 713 E1, E2, and E3 ubiquitin ligases, deubiquitinases, and control genes and contains 2,852 guide RNAs expressed in pXPR003 backbone. The virus for the library was produced as previously described(27, 28). 10% (v/v) of BISON CRISPR library was added to 6 x 10^6 HEK293TCas9 cells and transduced (2400rpm, 2h, 37°C). Eight days post infection cells were treated with 1μM CC-885 or DMSO for 4h and subjected to flow sorting (5% GSPT1-GFP/mCherry high and 5% GSPT1-GFP/mCherry low). Sorted cells were harvested by centrifugation and subjected to direct lysis, library preparation, sequencing and analysis as specified before Refs (27, 28) in main manuscript.

Human proteomics

Proteomics on native 293T cells or 293T cells overexpressing GSPT1, or GSPT1^{G575N}:

293T cells were treated with DMSO or 1μM CC-885 for 18 hours before harvesting cells, washing twice in cold PBS and freezing at -80ºC. Cell pellets were lysed at 4ºC in a solution containing 8M urea, 50mM Tris-HCl pH8.0, 150mM NaCl, 1mM EDTA, 2μg/μl Aprotinin (Sigma-Aldrich), 10 μg/μl Leupeptin (Roche), and 1mM phenylmethylsulfonyl fluoride (PMSF) (Sigma). Protein concentration was determined using a bicinchoninic acid (BCA) protein assay according to manufacturer's instructions (Pierce). Samples were first reduced with 5mM (DTT) for 45 min at

room temperature (RT). Samples were subsequently alkylated with 10 mM iodoacetamide for 30 min at room temperature in the dark. Urea concentration was reduced to 2M with 50mM Tris-HCl, pH8. Samples were pre-digested for 2h at 23ºC with Endoproteinase Lys-C (Wako Laboratories) at an enzyme-to-substrate ratio of 1:50. Samples were digested overnight at 23ºC with sequencing grade trypsin (Promega) at an enzyme-to-substrate ratio of 1:50. Subsequent to the digestion procedure, samples were acidified with 100% formic acid to a final concentration of 1% formic acid. Acidified samples were then desalted using a 200mg reversed-phase tC18 Sep-Pak cartridge (Waters). In brief, cartridges were conditioned with 3 mL of 100% MeCN, 3ml of 50% MeCN/0.1% FA, and 4x with 3ml of 0.1% TFA. After cartridge equilibration, samples were loaded and washed 3x with 3ml of 0.1% TFA and 1x with 3ml of 1% FA. Samples were eluted twice with 1.5ml of 50% MeCN/0.1% FA. Peptides were frozen at -80ºC and dried using a SpeedVac concentrator. Peptide concentration was then determined using a BCA assay as previously described. 100μg of peptide per sample was then aliquoted, frozen, dried using a SpeedVac concentrator, and stored at -80ºC

TMT labeling of peptides

100μg of desalted peptides aliquots were labeled with TMT 11-plex isobaric mass tagging reagents (Thermo Fisher Scientific). Peptides were resuspended in 20μl of 50 mM HEPES, pH 8.5, at a 5mg/ml total concentration. Samples were then labeled with 5μl of their respective TMT reagent at a concentration of 20μg/μl in anhydrous MeCN in a randomized scheme. Samples were incubated at 25ºC for 1 hour while shaking. Following evaluation of TMT labeling efficiency and ensuring evenly balanced peptide input from each sample, the labeling reaction was

quenched with 2µl of 5% hydroxylamine at room temperature for 15 min while shaking. TMT labeled samples were combined, dried to completion, reconstituted in 1ml of 0.1% FA, and desalted on StageTips or 200 mg SepPak columns as described above.

Basic Reverse Phase (bRP) Fractionation

The TMT labeled samples (1.1mg total peptide material) were fractionated. through high pH reversed-phase chromatography (bRP) using a 4.6 x 250 mm Zorbax 300 Extend C18 column (Agilent) on an Agilent 1260 series high pressure liquid chromatography (HPLC) system. bRP solvent A was made in an aqueous solution of 5mM of Ammonium Formate and 2% ACN (pH 10), and bRP Solvent B was made in a solution of 5mM Ammonium Formate and 90% ACN (pH 10). Samples were reconstituted in 900µL of bRP solvent A and injected into the instrument Fractionation proceeded at a flow rate of 1ml/min and separated using a 96 min gradient. The fractionation gradient proceeded with a bRP solvent B composition of: 0% to 7 minutes, 16% at 13 minutes, 40% at 73 minutes, 44% at 77 minutes, and 60% from 82 minutes to the end of the gradient. Fractions were collected in a 96- deep well plate (GE Healthcare) and concatenated in a 24-fraction scheme as previously described (Reference 41 in main manuscript). Pooled fractions were dried to completeness using a SpeedVac concentrator.

:

Liquid chromatography and mass spectrometry

Each sample fraction was resuspended in 3% MeCN/0.1% FA at a concentration of 1μg/μl and analyzed by nanoflow liquid chromatography tandem mass spectrometry (LC-MS/MS). One microgram of each fraction was injected onto a Q Exactive Plus Hybrid Quadrupole-Orbitrap Mass Spectrometer (Thermo Fisher Scientific) coupled to a Proxeon Easy nLC 1000 (Thermo Fisher Scientific). Samples were loaded onto a 360μm outer diameter × 75μm inner diameter microcapillary column with an integrated emitter tip heated to 50 ºC. The capillary column was packed in-house with ReproSil-Pur C18-AQ 1.9 μm beads (Dr. Maisch GmbH) to an approximate length of 24 cm. Samples were separated through acidic reverse-phase chromatography. Solvent A was composed of 3% ACN and 0.1% FA. Solvent B was composed of 90% ACN and 0.1% FA. Chromatography proceeded with a gradient profile as such (% solvent B to minutes): 2% at the 0-minute mark, 6% at 1 min, 30% at 85 min, 60% at 94 min, 90% at 95 min, 90% at 100 min, 50% at 101 min, and 50% at 110 min. A flow rate of 200nl/min was used for the first 100 min, with the last 10 minutes at 500nl/min.

The Q Exactive Plus mass spectrometer was operated in data-dependent mode with HCD MS/MS unimolecular dissociation. MS1 was set to a resolution of 70,000, an AGC target of 3e6, maximum injection time of 5 ms, and a scan range of 300 to 1800 m/z. MS2 was set to a resolution of 35,000, an AGC target of 5e4, a 120 ms max injection time, an isolation window of 0.7 m/z, fixed first mass at 100.0 m/z, HCD normalized collision energy of 30, and a top 12 peak duty cycle. Peptide match was set to preferred. Charge exclusion was enabled for charge states that were unassigned, 1 and >6. A dynamic exclusion time was set to 20 seconds.

Data Analysis

All data were analyzed using Spectrum Mill version BI.07.04.210 (Agilent Technologies, Broad Institute of MIT & Harvard). Similar MS/MS spectra acquired on the same precursor m/z within +/- 60 s were merged. MS/MS spectra were excluded from searching if they were not within the precursor MH+ range of 750- 6000 Da or if they failed the quality filter by not having a sequence tag length >0.

All spectra were allowed +/- 20 ppm mass tolerance for precursor and product ions, 30% minimum matched peak intensity, and “trypsin allow P” enzyme specificity with up to 4 missed cleavages. The fixed modifications were carbamidomethylation at cysteine, and TMT-full Lys only mix. Variable modifications included oxidized methionine and N-terminal protein acetylation. A variable modification mass shift window was set from 0 to 70 Da. MS/MS spectra were searched against a 2014 UniProt human database with common contaminants added (“UniProt.human.20141017.RNFISnr.150contams”).

Individual spectra were automatically designated as confidently assigned using the Spectrum Mill autovalidation module. Specifically, a target-decoy based false-discovery rate (FDR) scoring threshold criteria via a two-step auto threshold strategy at the spectral and protein levels was used. First, peptide mode was set to allow automatic variable range precursor mass filtering with score thresholds optimized to yield a spectral level FDR of of 1.2%. Second, protein polishing mode was used to reach a target protein FDR of 0% and eliminate unreliable protein-level identifications by un-validating marginal PSMs.

The protein summary was generated using “subgroup top grouping”, and ratio data was calculated as each TMT reporter ion intensity over the mean of the two control replicates (native 293T cells treated with DMSO). Spectra were filtered from quantitation by excluding spectra with a precursor

ion purity of <50%. TMT10 reporter ion intensities were corrected for isotopic impurities in the Spectrum Mill protein/peptide summary module using the afRICA correction method. The protein report was filtered such that each protein was identified using at least 2 unique peptides, quantified with a reporter ion intensity detected in every channel and had at least 2 ratios measured. A list of human keratins was also filtered from analysis. Subsequent statistical analysis was done using R / Protigy.

Mouse proteomics

Cells were lysed by addition of lysis buffer (8 M Urea, 50 mM NaCl, 50 mM 4-(2-hydroxyethyl)-1-piperazineethanesulfonic acid (EPPS) pH 8.5, Protease and Phosphatase inhibitors) and manual homogenization by 20 passes through a 21-gauge (1.25 in. long). Bradford assay was used to determine the final protein concentration in the clarified cell lysate. 50 µg of protein for each sample was reduced, alkylated and precipitated using methanol/chloroform as previously described(44) and the resulting washed precipitated protein was allowed to air dry. Precipitated protein was resuspended in 4 M Urea, 50 mM HEPES pH 7.4, followed by dilution to 1 M urea with the addition of 200 mM EPPS, pH 8. Proteins were first digested with LysC (1:50; enzyme:protein) for 12 h at RT. The LysC digestion was diluted to 0.5 M Urea with 200 mM EPPS pH 8 followed by digestion with trypsin (1:50; enzyme:protein) for 6 h at 37 °C. Sample digests were acidified with formic acid to a pH of 2-3 prior to desalting using C18 solid phase extraction plates (SOLA, Thermo Fisher Scientific). Desalted peptides were dried in a vacuum-centrifuged and reconstituted in 0.1% formic acid for LC-MS analysis.

Data were collected using a TimsTOF Pro2 (Bruker Daltonics, Bremen, Germany) coupled to a nanoElute LC pump (Bruker Daltonics, Bremen, Germany) via a CaptiveSpray nano-electrospray source. Peptides were separated on a reversed-phase C_{18} column (25cm25 cm x 75 µm ID, 1.6 µM, IonOpticks, Australia) containing an integrated captive spray emitter. Peptides were separated using a 50 min gradient of 2 - 30% buffer B (acetonitrile in 0.1% formic acid) with a flow rate of 250 nL/min and column temperature maintained at 50ºC50 ºC.

DDA was performed in Parallel Accumulation-Serial Fragmentation (PASEF) mode to determine effective ion mobility windows for downstream diaPASEF data collection. The ddaPASEF parameters included: 100% duty cycle using accumulation and ramp times of 50 ms each, 1 TIMS-MS scan and 10 PASEF ramps per acquisition cycle. The TIMS-MS survey scan was acquired between 100 – 1700 *m/z* and 1/k0 of 0.7 - 1.3 V.s/cm^2. Precursors with 1 – 5 charges were selected and those that reached an intensity threshold of 20,000 arbitrary units were actively excluded for 0.4 min. The quadrupole isolation width was set to 2 *m/z* for *m/z* <700 and 3 *m/z* for *m/z* >800, with the *m/z* between 700-800 *m/z* being interpolated linearly. The TIMS elution voltages were calibrated linearly with three points (Agilent ESI-L Tuning Mix Ions; 622, 922, 1,222 *m/z*) to determine the reduced ion mobility coefficients ($1/K_0$). To perform diaPASEF, the precursor distribution in the DDA *m/z*-ion mobility plane was used to design an acquisition scheme for DIA data collection which included two windows in each 50 ms diaPASEF scan. Data was acquired using sixteen of these 25 Da precursor double window scans (creating 32 windows) which covered the diagonal scan line for doubly and triply charged precursors, with singly charged precursors able to be excluded by their position in the m/z-ion mobility plane. These precursor isolation windows were defined between 400 - 1200 *m/z* and 1/k0 of 0.7 - 1.3 V.s/cm^2.

LC-MS data analysisGlobal quantitative proteomics on mouse cells expressing Crbn variants: Data analysis

The diaPASEF raw file processing and controlling peptide and protein level false discovery rates, assembling proteins from peptides, and protein quantification from peptides was performed using directDIA analysis in Spectronaut 14 (Version 15.5.211111.50606, Biognosys). DirectDIA mode includes first extracting the DIA data into a collection of MS2 spectra which are searched using Spectronaut's Pulsar search engine. The search results are then used to generate a spectral library which is then employed for the targeted analysis of the DIA data. MS/MS spectra were searched against a Uniprot mouse database (September 2020). Database search criteria largely followed the default settings for directDIA including: tryptic with two missed cleavages, fixed carbomidomethylation of cysteine, and variable oxidation of methionine and acetylation of protein N-termini and precursor Q-value (FDR) cut-off of 0.01. Precursor quantification was performed using MS1 areas, cross run normalization was set to automatic and imputation strategy was set to no imputation. Proteins with poor quality data were excluded from further analysis (summed abundance across channels of <100 and mean number of precursors used for quantification <2). Protein abundances were scaled using in-house scripts in the R framework (45) and statistical analysis was carried out using the limma package within the R framework (46) (References 45 and 46 in main manuscript).
